# The net electrostatic potential and hydration of ABCG2 affect substrate transport

**DOI:** 10.1038/s41467-023-40610-5

**Published:** 2023-08-18

**Authors:** Tomoka Gose, Heather M. Aitken, Yao Wang, John Lynch, Evadnie Rampersaud, Yu Fukuda, Medb Wills, Stefanie A. Baril, Robert C. Ford, Anang Shelat, Megan L. O’Mara, John D. Schuetz

**Affiliations:** 1 Department of Pharmacy and Pharmaceutical Sciences, St. Jude Children’s Research Hospital, 262 Danny Thomas Place, Memphis, TN 38105 USA; 2 Australian Institute of Bioengineering and Nanotechnology, The University of Queensland, Australia, Cnr College Rd & Cooper Rd, St Lucia, QLD 4072 Australia; 3 Center for Applied Bioinformatics, St Jude Children’s Research Hospital, 262 Danny Thomas Place, Memphis, TN 38105 USA; 4 School of Biological Sciences, The University of Manchester, Oxford Road, Manchester, M13 9PL UK; 5 Department of Chemical Biology and Therapeutics, St. Jude Children’s Research Hospital, 262 Danny Thomas Place, Memphis, TN 38105 USA

**Keywords:** Membrane proteins, Protein structure predictions, Permeation and transport, Computational biophysics

## Abstract

ABCG2 is a medically important ATP-binding cassette transporter with crucial roles in the absorption and distribution of chemically-diverse toxins and drugs, reducing the cellular accumulation of chemotherapeutic drugs to facilitate multidrug resistance in cancer. ABCG2’s capacity to transport both hydrophilic and hydrophobic compounds is not well understood. Here we assess the molecular basis for substrate discrimination by the binding pocket. Substitution of a phylogenetically-conserved polar residue, N436, to alanine in the binding pocket of human ABCG2 permits only hydrophobic substrate transport, revealing the unique role of N436 as a discriminator. Molecular dynamics simulations show that this alanine substitution alters the electrostatic potential of the binding pocket favoring hydration of the transport pore. This change affects the contact with substrates and inhibitors, abrogating hydrophilic compound transport while retaining the transport of hydrophobic compounds. The N436 residue is also required for optimal transport inhibition of ABCG2, as many inhibitors are functionally impaired by this ABCG2 mutation. Overall, these findings have biomedical implications, broadly extending our understanding of substrate and inhibitor interactions.

## Abstract (web summary)

ABCG2, an ATP-binding cassette transporter, extrudes hundreds of hydrophilic and hydrophobic compounds from cells, playing roles in xenobiotic clearance or multidrug resistance in cancer. Gose et al provide key insights into ABCG2 substrate selection.

## Introduction

ABCG2 is a medically-relevant ATP-binding cassette (ABC) transporter. In cancer cells, it modifies the cytotoxic response to many structurally diverse and mechanistically-unrelated chemotherapeutic drugs^1^. ABCG2 also plays a key role in protecting hematopoietic stem cells from low-oxygen environments and cytotoxins^2,3^. Further, genetic variation of *ABCG2* is associated with increased risk of gout and hyperuricemia due to ABCG2 transport of the endogenous substrate uric acid^4^. Understanding the factors contributing to the interactions between ABCG2 and its substrates/inhibitors will provide a basis for the rational development of advanced therapeutics. Our understanding of the molecular interactions that ABCG2 employs to recognize inhibitors and substrates has been significantly advanced by cryo-EM structural studies^5–10^. These studies were crucial in identifying the functional role of amino acid F439 as a molecular “clamp” that is used in vitro and in live cells to actively engage substrates and inhibitors in the ABCG2 binding pocket^11^. Although inhibitors and substrates enter the same binding pocket, the substrate/inhibitor-bound ABCG2 cryo-EM structures reveal that substrates and inhibitors may be distinguished by differences that arise in part from the size of the compound: inhibitors occupy a larger volume and have a greater number of contact points within the binding pocket^7^. Like ABCB1^12^, there is overlap in substrate and inhibitor binding contacts in ABCG2^6,7,9^.

One conundrum among the five ABCG transporter family members (ABCG1, ABCG2, ABCG4, ABCG5, and ABCG8), is that ABCG2’s broad substrate specificity includes the ability to transport both hydrophobic and hydrophilic substrates. Human ABCG2 harbors two unique phylogenetically-conserved polar residues, T435 and N436, in the binding pocket^13^. These two residues have been speculated to contribute to ABCG2 hydrophobic and hydrophilic substrate transport in vivo. Cryo-EM structural studies reveal that the polar residue, N436, interacts with some substrates. When ABCG2 harboring an N436A substitution is purified and reconstituted into proteoliposomes, the substrate-stimulated ATPase activity and transport are impared^7,9^.

Here we show *in cellulo* that the substitution of N436A does not abolish the function of ABCG2, but profoundly impacts substrate selection. The previously published cryo-EM structures show that the evolutionarily conserved N436 residue in the ABCG2 binding pocket plays a unique role in substrate transport. Using a potent combination of mutagenesis, in cellulo functional assays, molecular dynamics simulations (MDS), and structural modeling, we show that this conserved polar residue in ABCG2 has pleiotropic effects on the interactions with substrate and inhibitor.

## Results

### Identification of the binding pocket residues required for thermal stabilization and transport of Hoechst 33342

We first examined how ATP binding altered ABCG2 substrate interaction using thermal stability experiments. ATP treatment of ABCG2-WT expressing membranes produced a 2.5 °C increase in the T_m_ compared to untreated membranes, indicating an ATP-dependent thermal stabilization (Supplementary Fig. 1a). Next, we used cells expressing either ABCG2-WT or ABCG2-K86M (a mutant capable of binding, but not hydrolyzing ATP^14^) and performed a cellular thermal shift assay^15^ with or without depletion of intracellular ATP by non-toxic 2-deoxy-d-glucose and sodium azide treatment. Depletion of intracellular ATP produced reductions in the T_m_ for both ABCG2-WT and ABCG2-K86M, and since this mutant is catalytically inactive, the ATP interaction alone seems sufficient to produce thermal stabilization (Supplementary Fig. 1b). Based on our previous studies^11^, we hypothesized that substrates might thermally stabilize ABCG2. The ABCG2 substrate, Hoechst 33342, thermally stabilized ABCG2-WT in a dose-dependent manner in the absence of ATP (Supplementary Fig. 1c). We did not test it in the presence of ATP because an ATP-bound ABC transporter would bind substrate poorly as it is in a low affinity state^16^. The reduced affinity for substrate in an ATP-bound state was confirmed by comparing the findings for Hoechst 33342 interaction with ABCG2-K86M and ABCG2-E211Q (catalytically inactive mutants) in the absence and presence of ATP (Supplementary Fig. 1c, d). These findings provided the basis for our decision to omit ATP in our experiments assessing the impact of ABCG2 binding pocket mutations on Hoechst 33342 interaction.

**Fig. 1:**
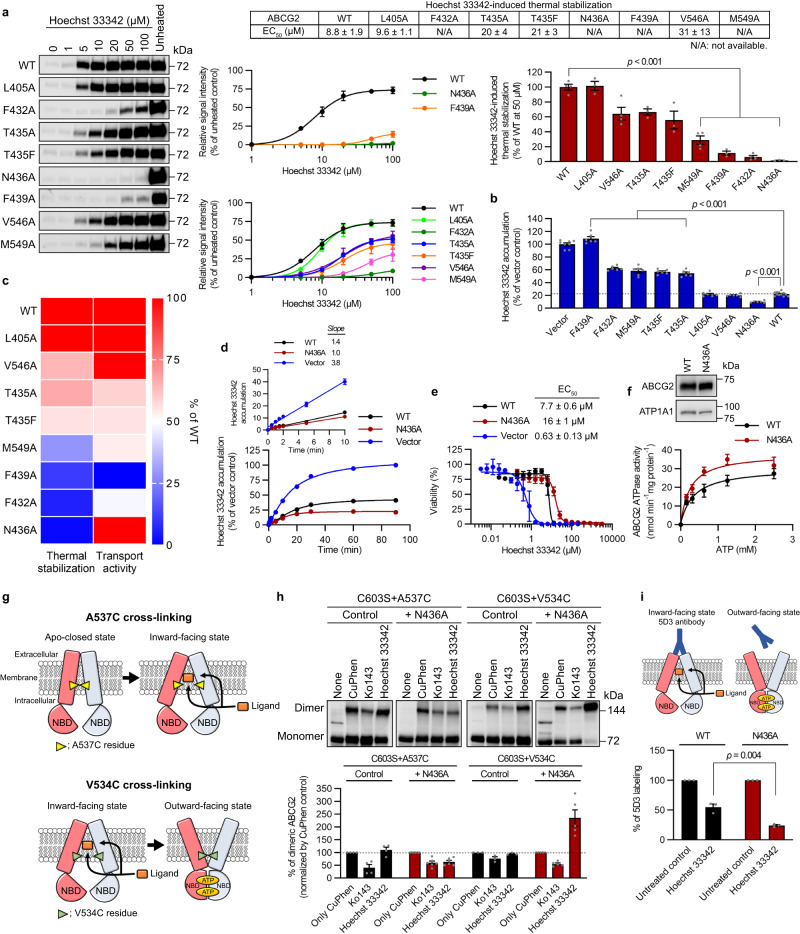
Characterization of the impact of ABCG2 binding pocket mutations on Hoechst 33342 thermal stabilization and transport. **a** Hoechst 33342-induced thermal stabilization of wild-type (WT) and ABCG2 mutants. Representative ABCG2 western blots (left), densitometry quantification (middle, mean ± SEM) from independent experiments (*n* = 4 (WT, F432A, V546A, and M549A) or *n* = 3 (all others)), and the EC_50_ values for Hoechst 33342 thermal stabilization (top, mean ± SEM). Signal intensities relative to ABCG2-WT at 50 μM Hoechst 33342 (right, mean ± SEM. *P* < 0.001 vs. WT). **b** Intracellular Hoechst 33342 levels in the MEFs expressing ABCG2 are shown as percentage of vector control (mean ± SEM, representative of three independent experiments. *P* < 0.001 vs. WT). **c** Heatmap shows the relative levels of Hoechst 33342 thermal stabilization (**a** (right)) and transport activity (**b**), with red being 100% and blue being no thermal stabilization or transport activity. **d** Relative intracellular Hoechst 33342 levels in the MEFs normalized to vector control at 90 min (mean ± SEM, representative of three independent experiments). **e** Hoechst 33342 cytotoxicity in the MEFs expressing ABCG2-WT (black), ABCG2-N436A (red), or vector control (blue) (mean ± SEM of four independent experiments). **f** Vanadate-sensitive ATPase activities for ABCG2-WT and ABCG2-N436A membranes quantified over a range of ATP concentrations (mean ± SEM of four independent experiments). Representative ABCG2 western blot (top). **g** A537C forms intermolecular disulfide bonds when ABCG2 is in the apo-closed state^8^. V534C forms intermolecular disulfide bonds when ABCG2 is in the nucleotide-bound outward-facing state. **h** CuPhen-induced crosslinking of the V534C or A537C monitored with non-reducing SDS-PAGE and western blot analysis of ABCG2. Upper band represents disulfide crosslinked ABCG2 (dimer; ~150 kDa). Representative ABCG2 western blots (top) and densitometry quantification (bottom, mean ± SEM) from independent experiments (*n* = 4 (C603S + A537C), *n* = 6 (C603S + A537C + N436A), *n* = 3 (C603S + V534C), or *n* = 5 (C603S + V534C + N436A)). **i** ABCG2-WT and ABCG2-N436A labeled with 5D3-A647 antibody in the presence of 50 μM Hoechst 33342 (mean ± SEM of three independent experiments. *P* = 0.004 vs. WT). All *P* values were calculated using two-tailed Student’s *t* test.

To investigate the importance of the contacts identified in ABCG2 cryo-EM structures to Hoechst 3342 thermal stabilization, we developed retroviral expression vectors containing variants of ABCG2 with binding pocket mutations (L405A, F432A, T435A, T435F, N436A, F439A, V546A, and M549A). Membrane vesicles were prepared from *Abcg2-*KO MEFs expressing these ABCG2 mutants. Isothermal shift assays were performed on the membranes by varying the Hoechst 33342 concentration at a fixed, empirically determined, optimal temperature (see “Methods”). Hoechst 33342 produced concentration-dependent stabilization of ABCG2-WT (Fig. 1a). The effect of the binding pocket residues on Hoechst 33342 thermal stabilization was not uniform and revealed interesting patterns: the alanine substitution of residues T435 in transmembrane helix 2 (TMH2), and V546 and M549 in TMH5, slightly reduced Hoechst 33342 thermal stabilization, suggesting that Hoechst 33342 still strongly or moderately bound these ABCG2 mutants. In contrast, alanine substitution of residues F432, N436, and F439 in TMH2 mostly prevented Hoechst 33342 thermal stabilization, suggesting a loss in Hoechst 33342 interaction (Fig. 1a).

In a peristaltic transport model such as that proposed for ABCG2^7^, transport is a dynamic process that requires the initial entry of substrate into the binding pocket, permeation and coordination of substrate, prior to an ATP-induced conformational change that powers efflux. We hypothesized that variants with binding site mutations that had the largest effect on the Hoechst 33342-induced thermal stabilization would also display impaired Hoechst 33342 transport. Hoechst 33342 cellular accumulation assays were used to determine the relationship between Hoechst 33342 thermal stabilization/interaction and transport. Expectedly, the F439A protein was not thermal stabilized by Hoechst 33342 and was incapable of transport (Fig. 1a, b). This is consistent with the proposed role of F439 as a molecular clamp that engages substrates^11^. The substitutions F432A and M549A also reduced Hoechst 33342 transport, which corresponded with their impaired ABCG2 thermal stabilization/interaction. Intriguingly, the N436A substitution markedly reduced Hoechst 33342 thermal stabilization/interaction with no loss in transport. Instead, ABCG2-N436A had enhanced Hoechst 33342 transport, as reflected by the greater reduction in the intracellular level of Hoechst 33342 (Fig. 1b). This was unexpected as purified ABCG2-N436A reconstituted into proteoliposomes has been reported to be incapable of estrone 3-sulfate transport^7^. The relationship between thermal stabilization/interaction and transport is depicted in the heatmap of the binding pocket mutants (Fig. 1c) and shows ABCG2-N436A is an outlier with improved transport and loss of the thermal stabilization/interaction.

### N436A enhances Hoechst 33342 transport and alters conformational response

The ABCG2-N436A substitution displayed markedly reduced intracellular Hoechst 33342 indicated by decreased steady-state accumulation (Fig. 1d). The difference between ABCG-WT and ABCG2-N436A was not due to a difference in Hoechst 33342 initial uptake, given that the initial rate of uptake was linear and the slopes virtually identical for ABCG2-WT and ABCG2-N436A (Fig. 1d). The steady-state level of Hoechst 33342 was twofold lower in ABCG2-N436A cells (Fig. 1d). Steady-state drug levels represent an equilibrium between uptake and efflux rates, which can be used to estimate the rate of drug efflux^17^. The twofold lower steady-state level of Hoechst 33342 in cells expressing ABCG2-N436A, therefore, indicates an estimated twofold increase in efflux (Fig. 1d). Direct monitoring of Hoechst 33342 efflux is not possible because of its avidity for binding to the minor groove of DNA, leaving little free Hoechst 33342 available for assessing efflux. This effect is also responsible for Hoechst 33342’s cytotoxicity, a property that we utilized as an additional approach to assess ABCG2 transport activity^18^. The Hoechst 33342 cytotoxicity assay showed that N436A-expressing cells exhibited a twofold greater Hoechst 33342 EC_50_ than that for ABCG2-WT cells, indicating strong protection compared to the empty vector expressing cells (Fig. 1e). The greater reduction in Hoechst 33342 cytotoxicity in ABCG2-N436A may be because Hoechst 33342 cytotoxicity occurs by a dual process of DNA binding and topoisomerase inhibition, both of which promote cytotoxic DNA damage. An investigation of the ATPase activity of ABCG2-WT and ABCG2-N436A showed that the ATP-concentration response curve for ABCG2-N436A shifted to the left, with a 5.7-fold lower EC_50_ value, whereas there was no significant difference in the maximum effect (E_max_) of ATP between WT (E_max_ = 48 +/− 19 nmol min^−1^ mg protein^−1^ and EC_50_ = 1.7 +/− 1.4 mM) and N436A (E_max_ = 40 +/− 6 nmol min^−1^ mg protein^−1^ and EC_50_ = 0.3 +/− 0.1 mM) (Fig. 1f). Consistent with the lower EC_50_ value of ATP (0.3 mM) for ABCG2-N436A, ABCG2-N436A was thermally stabilized by the non-hydrolyzable ATP analog AMP-PNP at lower concentrations than ABCG2-WT (Supplementary Fig. 2a). Higher substrate concentrations can inhibit ATPase activity^19^. As expected, Hoechst 33342 dose-dependently reduced ABCG2-WT ATPase activity in membranes with an IC_50_ of 35 μM (Supplementary Fig. 2b), consistent with the literature^20^, while membranes from cells expressing ABCG2-N436A appeared mostly refractory to Hoechst 33342 inhibition, consistent with our impaired Hoechst 33342 thermal stabilization results (Supplementary Fig. 2b and Fig. 1a).

We hypothesized that the ABCG2 conformational responses to Hoechst 33342 binding may be altered by N436A substitution. TMH5 of ABCG2 rotates between the apo-closed (inward-facing), and outward-facing nucleotide-bound states^8^ with two residues in TMH5, V534 and A537, capable of sensing native substrate-induced conformational changes. After cysteine substitution, these residues form cross-links when closely apposed in the presence of an oxidant, as depicted in Fig. 1g. Treatment of cells with the oxidant, copper phenanthroline (CuPhen), shows the extent of intermolecular ABCG2 dimer formation in intact cells as previously described^8^. We created cells harboring ABCG2 expression plasmids for either V534C or A537C substitutions along with the extracellular C603S substitution to prevent the formation of an intermolecular disulfide bond C603–C603’^8^. Cell lines that expressed these ABCG2 chimeras were as follows: ABCG2-WT or ABCG2-N436A and either V534C/C603S or A537C/C603S. In the presence of CuPhen, oxidation of the cysteines occurred at V534C when ABCG2 was in the outward-facing ATP-bound state and at A537C when ABCG2 was in the apo-closed state (see diagram Fig. 1g). As expected, the potent ABCG2 inhibitor, Ko143, impaired CuPhen-induced dimer formation by V534C in the context of ABCG2-WT, indicating that Ko143 blocked the formation of the outward-facing state (Fig. 1h). Hoechst 33342 did not block CuPhen-induced dimer formation by V534C (Fig. 1h). Unlike ABCG2-WT, the dimer formation by V534C in the context of ABCG2-N436A was markedly increased after treatment of cells with Hoechst 33342, suggesting an enhanced tendency towards the formation of an outward-facing state. To further delineate if the ABCG2-N436A conformation was altered, we employed 5D3, a conformationally sensitive ABCG2 antibody^21^, which strongly binds to the ECL3 when ABCG2 forms an inward-facing state^5^. Substrate binding typically promotes the formation of an outward-facing state, resulting in reduced or no effect of 5D3 binding. The stronger reduction in 5D3 binding for ABCG2-N436A by Hoechst 33342 compared to ABCG2-WT (Fig. 1i) suggested that ABCG2-N436A more readily transitioned from an inward-facing conformation to an outward-facing conformation after Hoechst 33342 binding.

### Genetic and structure-based insights into ligand interaction

ABCG1 is closely related to ABCG2, but prefers sterols whereas ABCG2 transports hydrophilic and hydrophobic substrates. An analysis of the ABCG1 and ABCG2 binding pocket regions that form the conserved TMH2 reveals that ABCG1’s binding pocket contains predominantly hydrophobic residues relative to ABCG2. The preponderance of variants in DNA sequence of ABCG2 from the Genome Aggregation Database (*GnomAD v3.1.1*) compared with ABCG1 suggests that ABCG2’s binding pocket is still undergoing evolutionary adaptation whereas ABCG1 is not (Supplementary Fig. 3). In accord with this, the genomic DNA sequences in ABCG2 appear more divergent than the translated peptide sequences. These highly conserved binding pocket regions forming TMH2 in ABCG1 and ABCG2 suggest the functional importance (Supplementary Fig. 3).

To assess the potential contribution of N436A in discriminating between substrates, atomistic molecular dynamics simulations (MDS) were used to investigate the initial binding interactions of three ABCG2 substrates displaying markedly different logP values: erlotinib (logP 2.3), Hoechst 33342 (logP 4.5), and tariquidar (logP 5.4), and compared to MDS of the inward-facing ABCG2 with no inhibitor or substrate present (apo-inward state). ABCG2-WT and two ABCG2 variants were studied: ABCG2-N436A and ABCG2-N436A-L405A. L405 was chosen based on our preliminary MDS data showing Hoechst 33342 interacting with the L405 residue of ABCG2-N436A protein, consistent with reports that some hydrophobic compounds interact with the L405 residue of ABCG2^6,9^. In accordance with experimental studies, these simulations were run using the highest resolution ATP-free inward-facing cryo-EM structure available with the inhibitor removed (PDB ID: 6ETI) and embedded in a solvated 80/20 POPC/Cholesterol bilayer. After equilibration, *n* = 3 replicate 300 ns simulations were produced for each system, giving 900 ns of combined simulations per system. To first assess protein stability in the MDS of the inward-facing conformation, the backbone RMSD of ABCG2 of each ABCG2 system was calculated, in the presence and absence of each compound (Supplementary Fig. 4). The time-dependent data for each independent simulation system (containing *n* = 3 concatenated replicate simulations) is provided as a Source data file. For each system, the RMSD plateaued within the first 50 ns of the simulation to a value consistent with thermal noise in an elongated membrane protein, indicating that ABCG2 did not undergo any large-scale conformational changes. Unless otherwise stated, the results discussed below were determined using statistical averages across the *n* = 3 replicate simulations and cluster analysis of the protein.

**Fig. 2:**
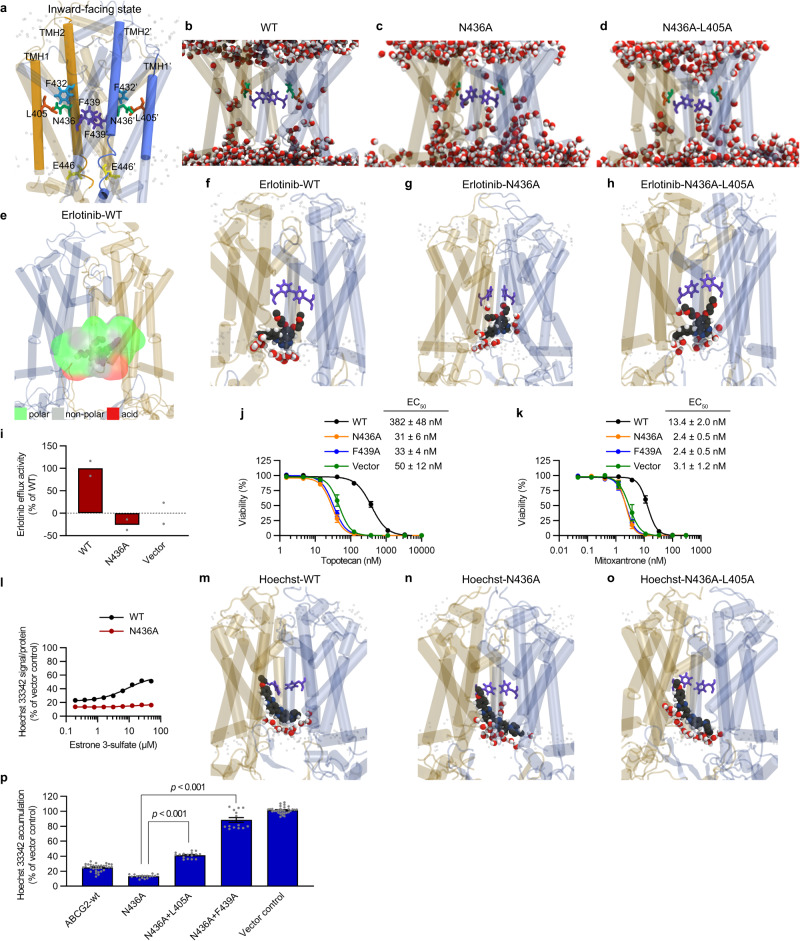
N436A substitution affects substrate interaction. **a** Initial conformation of the apo inward-facing ABCG2 structure, after equilibration, highlighting the location of key residues relative to the membrane. Phosphate headgroups are clear spheres, N436 is green, L405 is orange, F439 is purple, F432 is cyan, and E446 is yellow. Helices are in cartoon representation with TMH1 and TMH2 opaque. The individual monomers are colored gold (chain A) and blue (chain B). **b**–**d** MDS snapshots showing water (CPK coloring) permeation through ABCG2-WT (**b**), N436A mutant (**c**), and N436A-L405A mutant (**d**). **e**–**h** MDS snapshots showing the interaction of erlotinib with ABCG2. **e** ABCG2-WT with residues within 4 Å of erlotinib in a surface representation. Non-polar residues are grey, polar residues are green, and acidic residues are red. Erlotinib (dark grey space fill) bound structure in ABCG2-WT protein (**f**), N436A mutant (**g**), and N436A-L405A mutant (**h**). Water molecules within 4 Å of erlotinib are in CPK coloring. **i** Erlotinib efflux activity as percentage of ABCG2-WT (mean of one representative of three independent experiments). The MEFs expressing ABCG2-WT, ABCG2-N436A, or vector control were incubated with [^14^C]-erlotinib for 60 min. Cytotoxicity of topotecan (**j**) or mitoxantrone (**k**) in the MEFs expressing ABCG2-WT (black), ABCG2-N436A (orange), ABCG2-F439A (blue) or vector control (green) (mean ± SEM of independent experiments (*n* = 5 (**j**) or *n* = 4 (**k**))). **l** The MEFs were incubated with 0.5 μM Hoechst 33342 and estrone 3-sulfate (0.2–50 μM). Intracellular Hoechst 33342 levels in the MEFs expressing ABCG2 are shown as percentage of vector control (mean ± SEM of 3 independent experiments). **m-o** MDS snapshots showing ABCG2 with Hoechst 33342. Hoechst 33342 (dark grey space fill) bound structure in ABCG2-WT protein (**m**), N436A mutant (**n**), and N436A-L405A mutant (**o**). Water molecules within 4 Å of Hoechst 33342 are in CPK coloring. **p** Intracellular Hoechst 33342 levels in the MEFs expressing ABCG2-WT or ABCG2 mutants are shown as percentage of vector control (mean ± SEM of four independent experiments. *P* < 0.001 vs. N436A, two-tailed Student’s *t* test).

In assessing these MDS, we first considered the overall changes to apo-inward ABCG2 (Fig. 2a) on incorporation of the N436A and N436A-L405A mutations. In the membrane-embedded ABCG2-WT, MDS show solvent-accessible water wires within each individual monomer of the TMD, while the transport pore situated at the interface of the two TMD monomers is dehydrated (Fig. 2b). Substitution to N436A provided a favorable region for the permeation of water molecules, with 4 + /− 3 water molecules present in the binding pocket across the replicate (*n* = 3) 300 ns simulations in the apo inward-facing conformation (Fig. 2c and Supplementary Fig. 11a). This change was maintained in the double mutant N436A-L405A (Fig. 2d). In the first steps of substrate uptake, the substrate enters the transmembrane cavity and moves toward a minimum energy binding site, predicted to be located between the F439 clamps. While capturing the entire ABCG2 transport cycle is beyond the current capabilities of MDS, the ABCG2 6ETI cryo-EM structure provides a unique opportunity to gain insight into the initial stages of the permeation of these varied compounds as the F439 “clamping” residues are close together, sterically hindering the entry of substrates (Fig. 2a). This is unlike the tariquidar bound ABCG2 structure (PDB ID: 7NEQ), where tariquidar occupies the region between residues F439. While the docked orientation of tariquidar is different (see below and Supplementary Fig. 7) from previously published structural data (PDB ID 7NEQ), it is important to note that Kowal et al. show that tariquidar can adopt multiple conformations within the 7NEQ density map^9^. QM calculations on the conformation of tariquidar taken from the 7NEQ cryo-EM structure show that its geometry is highly constrained by an induced fit interaction with the protein during the transport cycle (Supplementary Data 1). While the cryo-EM conformation of tariquidar is not accessible using flexible docking approaches, several contact residues are conserved between MDS and the 7NEQ cryo-EM structure (Supplementary Fig. 7), suggesting the MDS samples intermediate conformations along the tariquidar transport pathway.

In studying the broad substrate specificity of ABCG2, it is important to note the concentration of polar residues lining the intracellular region of the transmembrane pore (Supplementary Fig. 5). This provides a favorable environment for initial coordination of hydrophilic substrates, such as erlotinib, in this region of the transport pore (Fig. 2e, f and Supplementary Fig. 5). Comparison of the ABCG2-WT and ABCG2-N436A shows the all-atom RMSD of erlotinib is extremely small across the 900 ns combined replicate simulation (0.9 + /− 0.2 Å and 1.1 + /− 0.2 Å, respectively), indicating the substrate is highly coordinated in both. Substitution to N436A significantly hydrates the erlotinib binding site (Fig. 2g), increasing the hydration number of the substrate from 17 + /− 7 in ABCG2-WT simulations (Fig. 2f) to 25 + /− 8 water molecules within 4 Å of the drug in ABCG2-N436A (Fig. 2g), further stabilizing this hydrophilic substrate (logP 2.3; erlotinib) in a position close to its initial uptake conformation (Fig. 2g, h and Supplementary Fig. 5). This reduces the propensity of erlotinib to permeate the transport pathway to the comparatively dehydrated F439 clamps (hydration number of 2 + /− 2 in the ABCG2-WT and 1 + /− 1 in the ABCG2-N436A simulations). Based on these simulations, we tested if N436 substitution affected transport of hydrophilic substrates. [^14^C]-labeled erlotinib was poorly exported by ABCG2-N436A, unlike ABCG2-WT (Fig. 2i). Furthermore, ABCG2-N436A was non-functional for topotecan (logP 2.0, Fig. 2j) and mitoxantrone (logP −0.5, Fig. 2k), in that it was unable to protect against their cytotoxicity. Monitoring of Hoechst 33342 intracellular accumulation after addition of the hydrophilic competitive substrate, estrone 3-sulfate (logP 2.6, Fig. 2l) indicated that as predicted, for ABCG2-WT, estrone 3-sulfate dose-dependently increased Hoechst 33342 accumulation, likely due to its competition with Hoechst 33342 for the N436 binding site. In contrast, accumulation of Hoechst 33342 by ABCG2-N436A was virtually unaffected by estrone 3-sulfate (Fig. 2l).

Hoescht 33342 has a logP of 4.5, consistent with a mildly hydrophobic substrate, yet contains three ionizable groups: the tetrahydropyrazine amine (pKa 7.87), and two benzoimidazole groups with pKa’s of 5.02 and 5.81^22^. At pH 7, the tetrahydropyrazine amine is likely to be protonated, resulting in an overall charge of +1 for Hoechst 33342^23^. Hoechst 33342 is positioned asymmetrically in the pore throughout the MDS, where it lies between TMH1 and TMH2 of a single monomer (Fig. 2m and Supplementary Fig. 8). The sole interaction with the opposing monomer is a single salt bridge between the tetrahydropyrazine cation and E446, on TMH2 of the opposing monomer (Supplementary Figs. 6 and 8). In ABCG2-WT simulations, the steric bulk of Hoechst 33342 prevents the interaction of N436 with TMH1, restricting its orientation such that N436 forms hydrogen-π interactions with F432, located one helical turn above N436 (Fig. 2m and Supplementary Figs. 6 and  8). Upon substitution to N436A, this interaction is lost, allosterically opening the F439-F439’ clamp (Supplementary Table 1 and Supplementary Fig. 9), which likely facilitates the permeation of Hoechst 33342 toward the center of the ABCG2 transport pore, between the F439 clamps (Fig. 2n and Supplementary Fig. 9). Substitution of L405 with alanine in the ABCG2-WT did not diminish either Hoechst 33342 interaction or transport for ABCG2-WT (see Fig. 1a, b). Yet, substitution of both N436A and L405A resulted in a marked loss of enhanced transport activity of ABCG2-N436A (Fig. 2p), indicating that this hydrophobic residue is functionally important for maximal ABCG2-N436A transport. MDS of this double mutation indicate that the drug changes position, occupying the void volume created by N436A-L405A substitution, below and adjacent to the F439 clamps (Fig. 2o and Supplementary Fig. 10b). We also show that, like the ABCG2-WT interaction, F439 is required for transport activity of ABCG2-N436A (Fig. 2p).

**Fig. 3:**
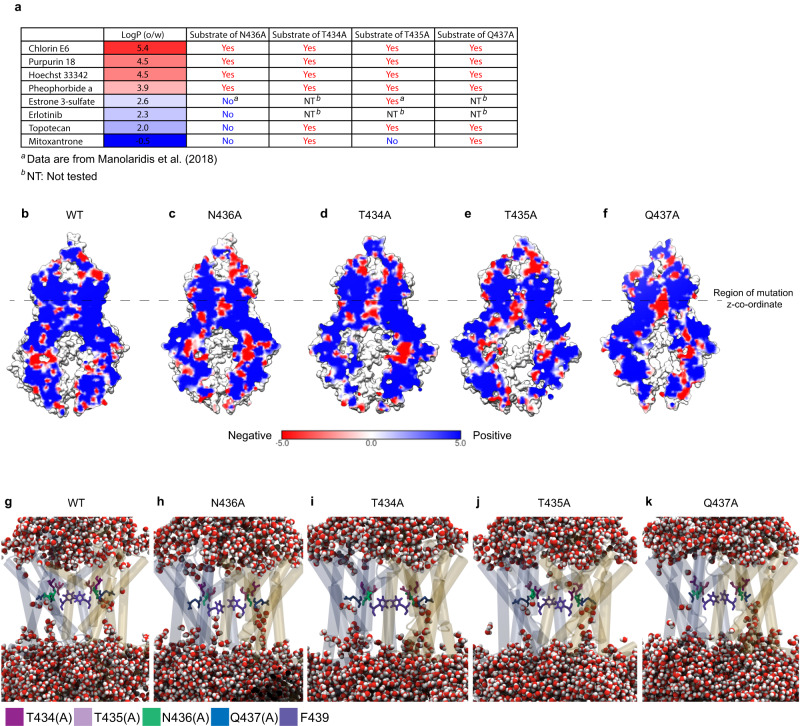
Substrate selectivity of polar residues (N436, T434, T435, and Q437) in the binding pocket of ABCG2. **a** ABCG2-N436A protein transports ABCG2-WT substrates with logP >3, but not ABCG2-WT substrates with logP <3. The other mutants have lower substrate selectivity than the N436A mutant. ABCG2 function was determined by substrate accumulation assay and cytotoxicity assay using the MEFs expressing ABCG2-WT, ABCG2-T434A, ABCG2-T435A, ABCG2-N436A, ABCG2-Q437A, or vector control. The intracellular accumulation of chlorin e6, purpurin 18, Hoechst 33342, and pheophorbide a was measured. The cytotoxicity data of topotecan and mitoxantrone in these mutants are from Fig. 2j, k, and Supplementary Fig. 11b, c. Experiments were repeated at least three times independently. Source data are provided as a Source Data file. The logP values were generated by molecular operating environment (MOE 2020.09). Compounds were neutralized in MOE at pH 7.4 and 2D descriptors were computed from the resulting structures. **b**–**f** Electrostatic potential maps, calculated using APBSmem, of **b** WT, **c** N436A, **d** T434A, **e** T435A, and **f** Q437A. **g**–**k** Water molecules (CPK coloring) within 4 Å of the protein after 15 ns of simulation for (**g**) WT, (**h**) N436A, (**i**) T434A, (**j**) T435A, and (**k**) Q437A.

Based on the MDS of Hoechst 33342, we hypothesized that ABCG2-N436A might prefer hydrophobic substrates. Using a cut-off of logP > 3, we next tested transport of additional hydrophobic substrates: chlorin e6, purpurin 18, and pheophorbide a (PhA). Unlike hydrophilic compounds, hydrophobic compounds were still readily transported by ABCG2-N436A (Fig. 3a). We further investigated the effect of other polar residues (T434, T435, and Q437) close to the N436 residue for substrate selectivity. The transport of hydrophobic substrates was not impaired by the alanine substitution of these polar residues (N436A, T434A, T435A, and Q437A). However, the transport of hydrophilic substrates in ABCG2-T434A, -T435A, and -Q437A was impaired to varying extents (Fig. 3a and Supplementary Fig. 11b, c). Protection against the cytotoxic effect of mitoxantrone’s cytotoxicity was lost for ABCG2-T435A and -N436A, whereas ABCG2-T434A and -Q437A retained some protction against its cytotoxicitty. Likewise, ABCG2-N436A was unable to protect against topotecan’s cytotoxicity, whereas ABCG2-T434A, -T435A, and -Q437A protected against the cytotoxic effect. These results indicate that N436A has the most strongest effect on hydrophilic substrate transport (Fig. 3a).

We investigated the role of these polar residues for the electrostatic potential and water penetration into the pore of ABCG2. Substitution to N436A introduced an area of negative electrostatic potential into the binding pocket (Fig. 3b, c). Alanine substitution of T434, T435, and Q437 had a similar effect on the electrostatic potential of the transmembrane region as the N436A substitution (Fig. 3b–f). However, in contrast to ABCG2-N436A, no water permeated into the binding cavity after 15 ns of MDS equilibration (Figs. 2c and 3g–k and Supplementary Fig. 11a) highlighting the dual nature of the role of N436A.

**Fig. 4:**
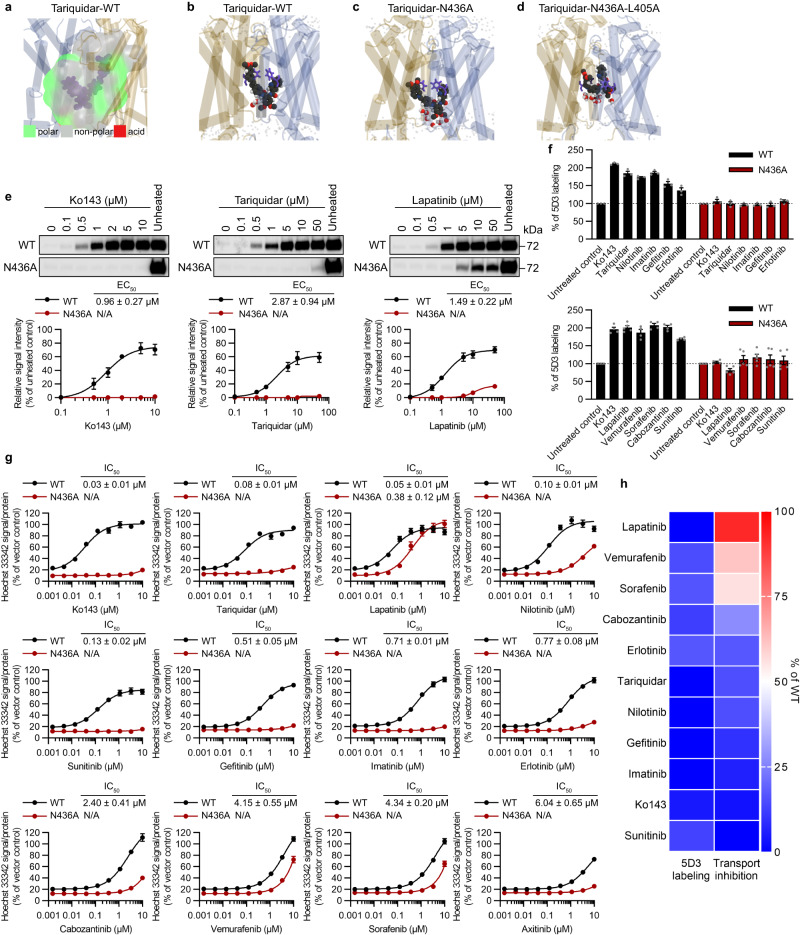
N436A substitution affects inhibitor interaction. **a**–**d** Representative snapshots from MDS of tariquidar interacting with ABCG2. **a** ABCG2-WT with residues within 4 Å of tariquidar are in surface representation. Non-polar residues are grey, polar residues are green, and acidic residues are red. The interaction of tariquidar (dark grey space fill) wiith ABCG2-WT (**b**), N436A mutant (**c**), and N436A-L405A mutant (**d**). Water molecules within 4 Å of tariquidar are shown in CPK coloring. **e** ABCG2 inhibitor-induced thermal stabilization of ABCG2-WT or ABCG2-N436A. Representative ABCG2 western blots (top) and densitometry quantification (bottom, mean ± SEM) from four independent experiments. N/A not available. **f** ABCG2-WT (black) and ABCG2-N436A (red) labeled with 5D3-A647 antibody in the presence of 5 μM Ko143, 10 μM tariquidar, or 10 μM kinase inhibitors (KIs) (mean ± SEM of three independent experiments (top graph) or 4 independent experiments (bottom graph)). **g** The inhibitory effect (IC_50_) of ABCG2 inhibitors for Hoechst 33342 transport in ABCG2-WT or ABCG2-N436A. Intracellular Hoechst 33342 levels in the MEFs expressing ABCG2 are shown as percentage of vector control (mean ± SEM of three independent experiments). N/A not available. **h** Heatmap showing the effect of N436A substitution for 5D3 labeling of ABCG2 with inhibitor (the data from **f**) and for transport inhibition at the concentration of maximum inhibition for ABCG2-WT (the data from **g**), with red (100%) being the effect of inhibitor for 5D3 labeling of ABCG2-WT protein, and the effect of transport inhibition for ABCG2-WT (blue panels indicate no effect of inhibitor for 5D3 labeling of ABCG2-N436A protein and no transport inhibition for ABCG2-N436A protein).

Erlotinib and tariquidar are also known to behave as ABCG2 inhibitors^24,25^. Unlike erlotinib, the hydrophobic inhibitor tariquidar binds deeper within the transmembrane domain, sandwiched between the F439 hydrophobic gates (Fig. 4a, b and Supplementary Figs. 7 and  8) where it forms transient π-stacking interactions, and is stabilized by surrounding hydrophobic residues within the pore (Fig. 4b and Supplementary Fig. 7). The N436A substitution slightly destabilizes the compound, increasing the all-atom RMSD of tariquidar from 0.7 + /− 0.2 Å in ABCG2-WT to 1.3 + /− 0.2 Å in the ABCG2-N436A simulations. In contrast to the hydrophilic substrate, erlotinib, the increase in hydration at the intracellular end of the pore resulting from the N436A substitution facilitates the permeation of tariquidar (logP 5.4) into the F439 clamps (Fig. 4c). On introduction of a double substitution, ABCG2-N436A-L405A, tariquidar permeates deeper between the F439 clamping residues (Fig. 4d and Supplementary Figs. 7 and 10), which suggests that it is adopting a substrate-like rather than inhibitor-like conformation. Substitution of both N436A and L405A was associated with a localized loss of helix-helix interactions between TMH1 and TMH2 which acted to widen the F439 clamps, increasing the Cα-Cα distance from 12.9 + /− 0.7 Å to 13.5 + /− 0.5 Å (Supplementary Table 1). This is associated with a reorientation of tariquidar and its intercalation between the gating F439 side chains, including transient observations of the drug adopting conformations similar to the constrained cryo-EM structure (PDB 7NEQ). Tariquidar is known to be transported, albeit slowly, by ABCG2^9^. We speculate that the ABCG2-N436A-L405A mutation may result in increased transport rather than inhibition by tariquidar (Fig. 4d and Supplementary Figs. 7 and 10).

### N436 is essential for efficacious inhibition

We next investigated the relationship between inhibitor-induced thermal stabilization and transport inhibition by N436A substitution. Hoechst 33342 uptake was used as a probe to determine the impact of transport inhibition. ABCG2 inhibitors, Ko143, tariquidar, and lapatinib, strongly and dose-dependently thermally stabilized ABCG2-WT (Fig. 4e). Thermal stabilization was unexpected as inhibitors reduced the thermal stability of ABCB1^15,26^. Intriguingly, the N436A substitution markedly reduced both inhibition and thermal stabilization; abolishing Ko143 and tariquidar inhibition and strongly attenuating lapatinib inhibition (Fig. 4e, g). The conformationally sensitive 5D3 antibody was used to evaluate if these inhibitors still promoted the formation of an inward-facing state which would be reflected by increased 5D3 reactivity^27^. As expected, for ABCG2-WT, all tested inhibitors increased 5D3 reactivity by at least 1.5-fold, suggesting that inhibitors generally immobilize ABCG2-WT in the inward-facing conformation (Fig. 4f). In contrast, for ABCG2-N436A, 5D3 reactivity was not uniformly increased by all KIs which suggested reduced capability of immobilizing ABCG2-N436A in the inward-facing conformation (Fig. 4f). We extended this analysis to determine the impact of the N436A substitution on ABCG2 transport inhibition by 9 additional kinase inhibitors (KIs). Strikingly, transport inhibition by all the KIs was impaired by the N436A substitution (Fig. 4g). The heatmap depicts how the N436 interaction is crucial for maximal inhibition and shows that the N436A substitution produced greater than a 50% loss in the inhibitory activity of Ko143, tariquidar, sunitinib, imatinib, gefitinib, nilotinib, and erlotinib (Fig. 4h). Unlike ABCG2-WT which had virtually no correlation between inhibitors’ hydrophobicity (logP) and transport inhibition (Supplementary Fig. 12a), the inhibitors’ hydrophobicity (logP) weakly correlated with transport inhibition in ABCG2-N436A (Supplementary Fig. 12b), supporting the idea that hydrophobicity contributes to the drug binding/recognition mechanism. This moderate correlation suggests there are other important factors beyond the scope of this work.

**Fig. 5:**
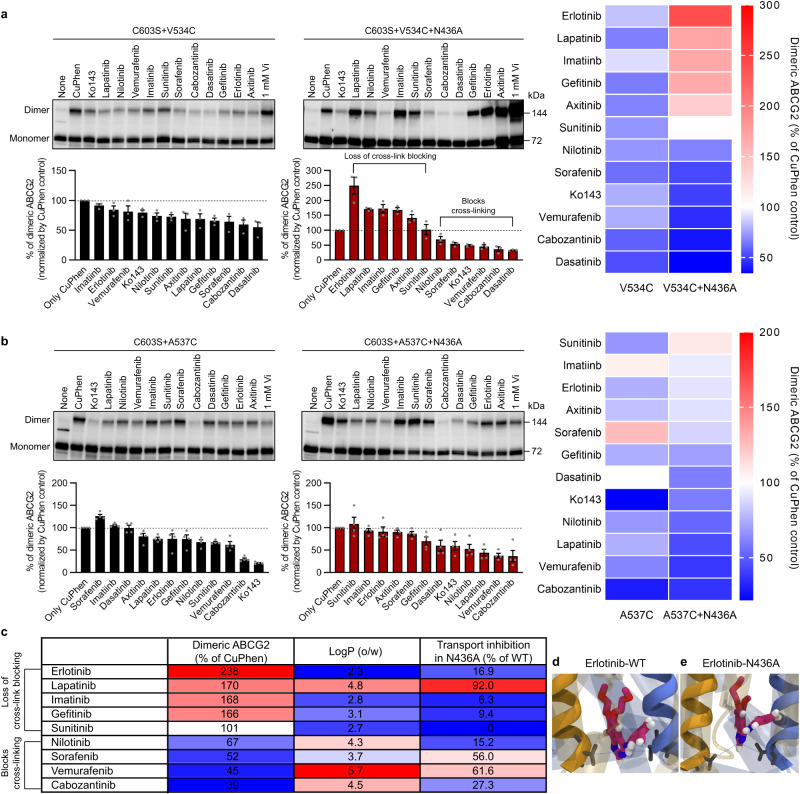
N436A substitution affects ABCG2 conformational response to inhibitors. **a**, **b** CuPhen-induced crosslinking of the V534C (**a**) or the A537C (**b**) monitored with non-reducing SDS-PAGE and western blot analysis of ABCG2. Sodium orthovanadate (Vi) locks ABCG2 into a nucleotide-bound state. Upper band represents disulfide crosslinked ABCG2 (~150 kDa). Representative ABCG2 western blots (top) and densitometry quantification (bottom, mean ± SEM) from independent experiments (*n* = 3 (**a**) or *n* = 4 (**b**)). The heatmap shows the effect of each inhibitor on the crosslinking (right). Blue and red indicate a decrease and increase, respectively, while white is no change when compared to CuPhen control. **c** Relationship between the effect of ABCG2 inhibitors for V534C cross-linking in ABCG2-N436A, transport inhibition in ABCG2-N436A, and logP values of ABCG2 inhibitors. **d**, **e** MDS snapshots of the interaction of erlotinib with ABCG2-WT (**d**) and ABCG2-N436A (**e**) indicate that erlotinib sits between V534 in ABCG2-WT yet above V534 in ABCG2-N436A.

We further investigated the conformational change of ABCG2-N436A produced by the KI using CuPhen-induced cross-linking assay (Fig. 5a, b). When ABCG2 is in the inward-facing state, the V534C and A537C residues are too distant to form an intermolecular disulfide bond^8^ (see diagram Fig. 1g). The dimer formation occurs at V534C when ABCG2 is in the outward-facing ATP-bound state, and this assay allows us to assess if inhibitors prevent conformational changes from an inward-facing to an outward-facing state. In the context of ABCG2-WT, the ABCG2 conformational response when engaging with KIs indicated that CuPhen-induced dimer formation by V534C was prevented (Fig. 5a). In the context of ABCG2-N436A, a majority of the KIs (erlotinib, lapatinib, imatinib, gefitinib, axitinib, and sunitinib) no longer restricted dimer formation by V534C (Fig. 5a). We interpret the loss of cross-link blocking by N436A substitution to indicate that these KIs no longer block the transition to an outward-facing ATP-bound state. Some of the KIs retained an ability to prevent V534C mediated dimerization for ABCG2-N436A (Fig. 5a). We further interrogated whether N436A substitution influenced inhibitor interaction with the binding pocket of ABCG2, by monitoring dimer formation through CuPhen-induced cross-linking at A537C which is reported to form in the apo-closed state (Fig. 5b). In the context of ABCG2-WT, most KIs readily blocked dimer formation by A537C, with carbozantinib being among the most effective. Some exceptions were sorafenib, imatinib, and dasatinib (Fig. 5b). In the context of ABCG2-N436A, a majority of the KIs still reduced dimer formation by A537C (Fig. 5b), suggesting that these KIs could enter and interact with the binding pocket of ABCG2-N436A. This finding suggests that although many inhibitors are able to enter the binding pocket of ABCG2-N436A, the inhibitors have an impaired ability to immobilize ABCG2-N436A in the inward-facing conformation. There seems to be a correspondence between the KI’s logP value and retention of blockade of ABCG2 dimer formation by V534C in the context of ABCG2-N436A (Fig. 5c). Importantly, only for ABCG2-N436A, there was a close correspondence between a lower logP value and the loss of crosslink formation (Fig. 5c). For ABCG2-WT, there was no discernable relationship between the KI’s logP value and transport inhibition (Supplementary Fig. 12).

MDS of the hydrophilic KI erlotinib interacting with ABCG2-WT show that erlotinib is situated between the opposing V534 residues, physically preventing crosslinking (Fig. 5d and Supplementary Fig. 8). In contrast, in the MDS of ABCG2-N436A erlotinib preferentially dwells ~7 Å deeper in the pore of the ABCG2-N436A compared to ABCG2-WT (Fig. 5e and Supplementary Fig. 9). In this deeper location, erlotinib is no longer positioned between the opposing V534 residues, facilitating crosslinking (Fig. 5a).

## Discussion

Our current studies using functional assays, mutagenesis, structure-based modeling, and MDS reveal factors accounting for ABCG2’s broad ability to readily transport both hydrophilic and hydrophobic substrates. Unlike other ABCG family members with binding pockets that are predominantly composed of non-polar residues (i.e., ABCG1), the mammalian ABCG2 subfamily exploits a phylogenetically conserved polar asparagine, N436. Substitution with alanine reveals the importance of N436 and other polar residues in the transmembrane pore to permit discrimination between hydrophilic and hydrophobic compounds, thereby providing fundamental insights into why ABCG2 transports more diverse compounds than other ABCG family members. The cross-linking and conformational antibody studies using the N436A substituted ABCG2 substantiated the conformational changes and adaptations that were revealed by the MDS and informed how a hydrophobic preferring ABCG2-N436A interacts differently with ligands from the ABCG2-WT. These findings sharply contrast with the general correspondence between loss of ligand thermal stabilization (ligand interaction) and transport for both hydrophobic and hydrophilic substrates that occurs with the alanine substitution for the conserved phenylalanine, F439^11^. Cross-linking studies suggest that ABCG2-N436A also has an enhanced ability to “switch”, induced by substrate, to the ATP-bound outward-facing conformation. MDS demonstrated that Hoechst 33342 repositioning in the transmembrane pore facilitates its transport. Importantly, an N436 interaction was also requisite for inhibitory activity of the majority of inhibitors, which concurrently lose their ability to thermally stabilize ABCG2-N436A and become less capable of immobilizing ABCG2-N436A in the inward-facing conformation that was revealed through cross-linking studies first employed by Orlando and Liao^8^.

The increased Hoechst 33342 transport observed for the N436A substitution was unexpected. Our studies suggest that N436 is a “selectivity switch” in that hydrophobic substrates (e.g., Hoechst 33342, chlorin e6, purpurin 18, and PhA) are not negatively impacted by N436A substitution, whereas hydrophilic substrates like topotecan, erlotinib, and mitoxantrone are not transported, which appears related to the change in the electrostatic potential and hydration of the pore. Previous findings with the hydrophilic estrone 3-sulfate showed that transport activity was lost when the N436A substitution was introduced^7^. Our findings showing estrone 3-sulfate has a reduced inhibitory effect on Hoechst 33342 transport by ABCG2-N436A highlights how fundamental this asparagine interaction is to transport by ABCG2-WT *in cellulo*. These studies revealed a previously unknown role for N436 in the coordination of Hoechst 33342 and other hydrophobic substrates, while underscoring an essential role for hydrophilic substrates like topotecan, mitoxantrone, erlotinib, and estrone 3-sulfate. MDS of ABCG2-N436A also suggested that the loss of the N436 polar side chain influenced Hoechst 33342 position in the pore and expanded the binding region (Supplementary Figs. 8b and 9b). This was further highlighted by transport studies that revealed additional hydrophobic contacts (e.g., L405) in ABCG2-N436A (but not ABCG2-WT) that supported ABCG2-N436A mediated Hoechst 33342 transport (Fig. 2p). We speculate that this would not have been discovered with purified ABCG2-N436A unless it was reconstituted into the exact membrane environment of cells. Notably, the formation of Hoechst 33342-induced inward-closed ATP-bound state of ABCG2-N436A underscores that the bound conformation of Hoechst 33342 during the transport cycle is markedly different from ABCG2-WT. The role of the electrostatic potential and water penetration into the pore was further revealed by the substitution of additional polar residues (T434A, T435A, and Q437A) in the binding pore which had no effect on hydrophobic substrate transport, but impaired hydrophilic substrate transport (Fig. 3a and Supplementary Fig. 11b, c). Although alanine substitution of these polar residues (T434A, T435A, N436A, and Q437A) had a similar effect on the electrostatic potential of the transmembrane region, it was notable that only the N436A substitution increased hydration of the transport pore, highlighting the unique impact of N436 residue (Fig. 3b–k). Interestingly, alanine substitution of adjacent threonine residues at positions 434 and 435 showed different effects for the cytotoxicity of topotecan and mitoxantrone (Supplementary Fig. 11b, c). We speculate that the differential effect of the alanine substitution among these polar residues and hydrophilic substrate transport is likely related to an as yet unquantifiable combination of the effect on the electrostatic potential and the movement of water into the pore. The differences in the effects of alanine substitution among these residues do not seem to be easily explained by the compounds physicochemical properties (volume, Van der Waals surface area, logP, H bond acceptor, and H bond donor) (Supplementary Fig. 11d).

ABCG2-N436A showed the loss of inhibitor-induced thermal stabilization along with an impairment in inhibition. This lost thermal stability simply cannot be generalized to all ABC transporters and their inhibitors. ABCB1 inhibitors like verapamil and tariquidar did not induce thermal stabilization^26^: these inhibitors reduced the ATP-induced thermal stabilization of ABCB1 but did not directly compete with ATP. Thermal stabilization studies of ABCG2 were conducted without exogenous nucleotide because of ABCG2’s low affinity for ligand when ATP is bound^16^. The reduction of ATP-induced thermal stabilization of ABCB1 by inhibitors is likely related to mechanistic differences between ABCG2 and ABCB1. TMD and NBD coupling for ABCB1 are not as strict as they are for the homodimer ABCG2. This seems consistent with ABCB1 being less susceptible to inhibitor-reduced ATPase activity^10^.

The water flow for ABCG2 has been posited to generally impact transport of all ABCG2 substrates using the behavior of one hydrophilic substrate, mitoxantrone^28^. Elimination of the ABCG2 hydrophobic di-leucine (L554, L555) valve by double alanine or cysteine mutations (L554A L555A and L554C L555C) enhanced water flow and reduced mitoxantrone transport. We show that substitution of the polar asparagine, N436, with a non-polar alanine generally eliminated transport of multiple hydrophilic substrates but retained transport of hydrophobic substrates, suggesting that a loss of a polar side chain retains proper valve function. MDS show that substitution of N436 with alanine alters the electrostatic potential of the pore and increases the permeation of water molecules near N436A. These findings suggest that the effect of N436A substitution is dependent on the properties of the substrate.

Many ABCG2 inhibitors were deleteriously impacted by the N436A substitution, showing profound reductions in their efficacy. This broad general effect on inhibitors suggested a common but key mechanism that negatively impacted inhibitors by loss of the polar N436 in ABCG2. The cryo-EM structure of ABCG2 with tariquidar shows multiple binding modes^9^ and one infers that this might occur with other inhibitors. Based on our studies, loss of the polar side chain N436 slightly increases the volume of the binding pocket and disrupts inhibitor interactions with multiple residues, thereby impeding their ability to coordinate and lock ABCG2 into an inward-facing conformation. Many of the inhibitors with impaired ability to block cross-linking at V534C were the least hydrophobic whereas the inhibitors that retained the ability to keep ABCG2-N436A in the inward-facing conformation were more hydrophobic, suggesting certain physicochemical properties of inhibitors are important. The hydrophobic inhibitors vemurafenib and lapatinib still inhibited ABCG2-N436A transport (Fig. 4g, h). While vemurafenib retained the ability to keep ABCG2-N436A in the inward-facing conformation, lapatinib no longer blocked the transition to an outward-facing ATP-bound state (Fig. 5a, c). We speculate that lapatinib has an alternative mode of inhibition, which appears to act by blocking the action of F439 through π-stacking and hydrophobic interactions with these so called “clamps”.

Our current studies feature a mechanistic account of how a conserved N436 substitution produced an unexpected gain in transport function, coupled with a loss in thermal stabilization/interaction. The increased Hoechst 33342 transport is likely related to untethering the substrate from the restraint of the transmembrane pore’s polar interactions and an increased propensity to form an ATP-bound outward-facing state revealed by the V534C cross-linking. For inhibitors, N436 appears to be a key anchor that enables many inhibitors to adopt a thermostable ligand-bound conformation within the binding pocket. Thus, the phylogenetic conservation of N436 seems to play a key role in ligand-based conformational transitions and substrate selection.

## Methods

### Chemicals

Hoechst 33342 was obtained from Invitrogen. Pheophorbide a (PhA), chlorin e6, and purpurin 18 were obtained from Frontier Scientific. PhA, chlorin e6, and purpurin 18 were dissolved in DMSO and aliquots protected from light at −20 °C. [^14^C]-erlotinib was obtained from Moravek Biochemicals. Ko143 was obtained from Enzo Life Sciences. Lapatinib and tariquidar were obtained from Selleckchem. Ko143, lapatinib, tariquidar, and other compounds were stored at −20 °C at 10 mM in DMSO.

### Plasmid construction and site-directed mutagenesis

All site-directed mutations were generated in the MSCV-human ABCG2-IRES-GFP expression plasmid using QuikChange II XL site-directed mutagenesis kit (Agilent Technologies, Santa Clara, CA).

### Cell culture and transduction

Mouse embryonic fibroblast (MEF) cell line was derived from *Abcg2* knockout (KO) mice that were obtained from the Sorrentino lab^3^. This cell line was reported previously^11^. The *Abcg2-*KO MEFs were engineered to express human ABCG2 (Uniprot: Q9UNQ0). Cells were grown in Dulbecco’s Modified Essential Medium (D-MEM) (Lonza, Basel, Switzerland) supplemented with 10% fetal bovine serum (Gibco, Grand Island, NY), penicillin (100 U/ml), streptomycin (100 µg/ml) (Gibco, Grand Island, NY), and l-glutamine (2 mM) (Gibco, Grand Island, NY) at 37 °C with 5% CO_2_. The *Abcg2-*KO MEF cell line was transduced with the retroviruses harboring cDNAs encoding either the mutant human ABCG2-K86M, E211Q, L405A, F432A, T434A, T435A, T435F, N436A, N436A + L405A, Q437A, F439A, N436A + F439A, V546A, M549A, C603S + V534C, C603S + V534C + N436A, C603S + A537C, or C603S + A537C + N436A. The HEK293 cell line was transduced with the retroviruses harboring cDNAs encoding the mutant ABCG2-N436A.

### Substrate accumulation assays

The intracellular accumulation of fluorescent Hoechst 33342, pheophorbide a, chlorin e6, and purpurin 18, ABCG2 substrates, was used to determine the function of ABCG2. For Hoechst 33342 accumulation assay, MEFs were seeded into a 96-well black clear bottom plate (Corning #3603) at 20,000 cells/well and 0.5 μM Hoechst 33342, and either Ko143 or the test inhibitor (0.002–10 μM) were added to the culture medium followed by incubation for 2 h at 37 °C in the dark. Thereafter, the medium was removed, and cells were washed twice with 200 μL ice-cold PBS and the fluorescence of Hoechst 33342 was then quantitatively determined with a Synergy H4 microplate reader (BioTek, Winooski, VT, USA) at an excitation wavelength of 355 nm and an emission wavelength of 460 nm. The accumulation of Hoechst 33342 was normalized to protein concentration as determined using the BCA Protein Assay Kit (Pierce, Rockford, IL, USA). The data were normalized by the proportion accumulated relative (%) to vector control. For the time-dependent Hoechst 33342 accumulation assay, 10 μM Hoechst 33342 was treated for different time periods (0.5, 1, 1.5, 2, 5, 10, 20, 30, 60, and 90 min). Relative intracellular Hoechst 33342 levels in the MEFs were normalized to vector control at 90 min. For the other fluorescent substrate accumulation assay, 0.5 μM pheophorbide a, 5 μM chlorin e6, or 5 μM purpurin 18 were treated for 1 h at 37 °C in the dark. The intracellular accumulation of pheophorbide a, purpurin 18, or chlorin e6 was measured at an excitation wavelength of 405 nm and an emission wavelength of 667 nm. For [^14^C]-labeled erlotinib accumulation assay, cells were seeded into a 24-well cell culture plate at 200,000 cells/well, and 0.1 μM [^14^C]-erlotinib was added to the culture medium followed by incubation for 1 h at 37 °C in the dark. Thereafter, the medium was removed and cells were washed twice with 1 mL ice-cold PBS. For the determination of intracellular [^14^C]-erlotinib, cells were lysed in 0.5 mL of 1 N NaOH, neutralized with 0.25 mL of 2 N HCl, and mixed with ProSafe FC+ liquid scintillation cocktail (Meridian Biotechnologies Ltd, Tadworth, UK). The total radioactivity was measured by liquid scintillation counter (Beckman Instrument, Inc., Columbia, MD).

### Plasma membrane preparation

MEF cell lines or HEK293 cell lines were pelleted and washed with PBS before suspension in a hypotonic buffer (0.5 mM Tris (pH 7.4), 0.1 mM EDTA, and protease inhibitors) and freezing. After gentle thawing, cells were pelleted by centrifugation, suspended in 2.5 mL of hypotonic buffer, and spun for 40 min in an SW32Ti swinging bucket rotor at 100,000 ×*g*. Pellets were resuspended into Homogenization Buffer (250 mM sucrose, 50 mM Tris, 250 µM CaCl_2_, and protease inhibitors) and homogenized with 50 strokes of a tight Dounce pestle. Following centrifugation at 500 × *g* for 5 min, the pellets were then resuspended in Homogenization Buffer and homogenized again before an additional 500 × *g* centrifugation step. The supernatants of each spin were pooled together and layered over a 5 ml 35% sucrose pad formed in 50 mM Tris (pH 7.4) and centrifuged in the SW32Ti rotor for 1.5 h at 100,000 × *g*. The interface was carefully removed and diluted with Dilution Buffer (50 mM Tris, (pH 7.4), 25 mM sucrose) and the crude membranes were pelleted at 100,000 × *g*. Pellets were resuspended in 25 mM Tris (pH 7.4) and incubated overnight at 4 °C on ice. These pellets were then passed 10× through a 27-gauge needle before protein quantification. The membranes were typically usually used immediately or stored at −80 °C for one thaw cycle.

### ATPase assays

For each sample, purified membrane vesicles (25 µg) from ABCG2-expressing HEK293 cells were dissolved in 50 µL total volume reaction buffer (40 mM MOPS (pH 7.0), 10 mM MgCl_2_, 50 mM KCl, 2 mM DTT, 0.5 mM EDTA, 5 mM sodium azide to block F-type ATPases, and 1 mM ouabain to inhibit Na^+^/K^+^ ATPase). Samples for Hoechst 33342 dose-response experiments were brought to the desired drug concentration (0.02, 0.2, 2, 10, 20, or 50 µM) or treated with vehicle and allowed to preincubate for 30 min at room temperature. ATP was freshly prepared in reaction buffer at twice the desired final concentration and brought to pH 7.0 and then incubated for 35 min at 37 °C. In ATP dose-response experiments, ATP was varied between 0 and 2.5 mM, while for Hoechst 33342 dose-response experiments, 2 mM ATP was used. Blanks were determined by cotreatment with 2 mM sodium vanadate. Reactions were stopped by immediate immersion in ice and centrifugation at 21,000 × *g* at 4 °C. In total, 50 µL of the supernatant was transferred to a 96-well plate and reacted with an equal volume of developing mix, a 1:1 mixture, made immediately before use, of 1% ammonium molybdate in water and freshly-made 6% ascorbate in 1 N HCl^29^. Plates were incubated at room temperature for 5 min for full color to develop and read at a wavelength of 800 nm. Quantitation was performed using phosphate standards diluted in reaction buffer in a range of 0–50 nmol, with standards and samples being treated identically.

### Cellular and membrane thermal shift assays

Cellular thermal shift assays were performed as described previously^11^. Human ABCG2-expressing membrane vesicles were prepared from MEFs lacking murine *Abcg2* and programmed to express human ABCG2 as described^11^. In some cases, human ABCG2-expressing membrane vesicles from HEK293 cells (purchased from Solvo Biotechnology, Budapest, Hungary) were used. The thermal shift assay was adapted according to ref. ^30^. Briefly, membrane vesicles (5 µg protein/20 µL final reaction volume) in 1x assay buffer (SB PREDIVEZ™ Reagent Kit for BCRP, SBPVR4, Solvo Biotechnology) were heated in a thermocycler for 3 min at various temperatures (37–78 °C) to establish a thermal denaturation curve. Samples were then treated with ice-cold PBS supplemented with NP-40 to a final concentration of 0.8%. Subsequently, ultracentrifugation (at 100,000×*g* for 20 min at 4 °C) was performed to precipitate the denatured proteins. The supernatant was subjected to immunoblot analysis using an ABCG2 antibody (Enzo Life Sciences Inc., BXP-53, 1:500). Based on an extrapolation from the thermal denaturation curve that produced 99% loss of protein in the supernatant, 65 °C was selected for wild-type (WT), 62 °C for L405A, T435F, and F439A, 64 °C for F432A and V546A, 67 °C for E211Q, N436A, and T435A, and 68 °C for K86M and M549A in the absence of MgATP to generate an isothermal dose-response curve. In the presence of MgATP (4 mM), 72 °C was selected for K86M and E211Q. To assess the ability of ligands to thermally stabilize ABCG2, membranes were first incubated with ligand for 60 min at 37 °C. Samples were then heated for 3 min. The signal intensity of the heated sample was normalized to the signal intensity of the unheated sample (WT and mutants) and the signal intensity was reported as % of either unheated control or % of ABCG2-WT. The ligand concentrations required for thermal stabilization are not true binding constants because they represent two processes: binding and thermal stabilization. To assess the ability of Hoechst 33342 to thermally stabilize ABCG2-K86M or E211Q in the presence of MgATP, membranes were first incubated with 4 mM MgATP for 30 min at 37 °C, and then Hoechst 33342 (1, 5, 10, 20, 50, and 100 μM) were added to the samples. Samples were incubated for 60 min at 37 °C and then heated for 3 min.

### Immunoblot analysis

Proteins were separated by SDS-PAGE (4–15% Criterion TGX Precast Gel, Bio-Rad), and transferred to a nitrocellulose membrane (Amersham Protran 0.45 μm NC, GE Healthcare). Immunoblot analysis was performed using primary antibody against ABCG2 (BXP-53, Enzo Life Sciences Inc., 1:500) and was subjected to further incubation with HRP-conjugated secondary antibody raised against rat IgG (Jackson ImmunoResearch Laboratories Inc., 1:10,000) as previously described^11^. Chemiluminescence signal was detected using LI-COR Odyssey Fc Imaging system (Image Studio 5.2). The bands were analyzed using LI-COR Image Studio Lite Ver 5.2 software for quantification of western blot signals. As a membrane marker, immunoblot analysis was performed using primary antibody against ATP1A1 (sodium potassium ATPase alpha 1, Novus Biologicals, Littleton, CO, 1:5000). As the secondary antibody, HRP-conjugated secondary antibody raised against mouse IgG (Jackson ImmunoResearch Laboratories Inc., 1:10,000) was used.

### Disulfide crosslinking

Disulfide crosslinking was adapted according to ref. ^8^. Briefly, the *Abcg2-*KO MEF cell line was transduced with the retrovirus harboring cDNAs encoding either the human mutant ABCG2-C603S+V534C, C603S+V534C+N436A, C603S+A537C or C603S+A537C+N436A. Other retroviral expression constructs were developed that harbored cysteine substitutions at either of the conformational sensing residues, V534 or A537 in a C603S background. Cells were incubated with either ligand (20 μM) or sodium orthovanadate (1 mM, Sigma Aldrich, S6508) as a positive control for 20 min. Copper phenanthroline was prepared by combining 500 mM copper(II) sulfate (Sigma Aldrich, 451657) in water with 500 mM 1,10-phenanthroline monohydrate (Sigma Aldrich, P9375) in ethanol, 6 mM (Cu^2+^)/(1,10-phenanthroline)_3_. Subsequently, the medium was replaced with medium containing the same ligands and 300 μM (Cu^2+^)/(1,10-phenanthroline)_3_. The copper phenanthroline oxidation proceeded for 45 min. Cells were then washed with PBS, resuspended in 25 mM Tris pH 8, 150 mM NaCl, 1% n-Dodecyl-β-d-Maltoside (ThermoFisher Scientific, 89902), 0.2% cholesteryl hemisuccinate (Cayman Chemical, 25698), and protease inhibitors (cOmplete EDTA-free Protease Inhibitor Cocktail, Roche, 05056489001) and incubated on a nutating rocker at 4 °C for 1 h to solubilize membranes. Samples were spun at 15,000 × g and the soluble fraction was mixed with SDS-PAGE loading buffer containing 40 mM EDTA (Fluka, 03690) and 40 mM N-ethyl maleimide (Sigma Aldrich, E1271). Samples were subjected to non-reducing SDS-PAGE, and ABCG2 was analyzed using monoclonal antibody BXP-53 (Enzo Life Sciences, ALX-801-036-C100) to monitor the extent of oxidant-induced crosslinking of the V534C or A537C mutant.

### Cytotoxicity assays

CellTiter-Glo® luminescent cell viability assay (Promega) was used to determine the cytotoxicity of Hoechst 33342, topotecan, and mitoxantrone using MEFs lacking murine *Abcg2* but expressing the human ABCG2-WT, N436A, F439A, T434A, T435A, Q437A, or vector control. Cells were seeded into a 96-well black clear bottom plate at 1000 cells/well and the cytotoxic compound was added to the culture medium followed by incubation for 3 days.

### 5D3 antibody shift assay for assessing the interaction between ABCG2 ligand and ABCG2-N436A

To determine ligand-induced ABCG2 conformational changes, we used the conformationally sensitive 5D3 antibody we developed^21^ that binds to extracellular loop 3 (ECL3) of ABCG2^5^. Briefly, Alexa-647 labeled 5D3 antibody binding to intact cells was measured by flow cytometry as described previously^27^. MEFs (3 × 10^5^ cells/sample) were preincubated with 5 μM Ko143, 50 μM Hoechst 33342, or 10 μM of the other ABCG2 ligands in Dulbecco’s PBS for 10 min at 37 °C before labeling with 0.5 μg/mL of 5D3 antibody (sc-18841, Santa Cruz Biotechnology, Santa Cruz, CA) for another 45 min at 37 °C. Cells were washed with PBS before incubation with the secondary antibody, anti-mouse IgG-Alexa-647 (A21235, ThermoFisher Scientific, 1:100) in 0.5% FBS/PBS for 30 min at 37 °C. Cells were washed with 0.5% FBS/PBS and resuspended in 0.5% FBS/PBS. To detect dead cells, cells are stained with propidium iodide (PI). Samples were run on a BD LSRFortessa flow cytometer (BD Biosciences) and analyzed using FlowJo v10 software. Alexa-647 labeled 5D3 antibody was excited with a 640 nm laser and emission was collected with a 670/30 filter. PI for dead cell exclusion was excited with a 562 nm laser and emission was collected with a 610/20 filter. 5D3 labeling was presented as the percent of the untreated control fluorescence measured in the presence of 5D3-A647 alone for each protein.

### Molecular dynamics simulations

Molecular dynamics simulations were performed using the Groningen Machine for Chemical Simulation engine, version 2019.4 (GROMACS 2019.4)^31^, with the GROMOS 54a7 force field^32^. The cryo-EM structure of ABCG2 in the ATP-free inward-facing (PDB ID 6ETI) conformation was used as the starting structure for simulations as it is the highest resolution ABCG2 structure available^6^. The residues missing from the cryo-EM structure were modelled using the ABCG2 Alphafold structural prediction (AlphaFold Protein Structure Database entry: Q9UNQ0)^33^. To investigate the impact of point mutations on ABCG2 in simulation, PyMOL (version 2.5.0, The PyMOL Molecular Graphics System, Schrödinger, LLC) was used to introduce a series of point mutations (N436A and N436A + L405A) to give starting coordinates for ABCG2-N436A and ABCG2-N436A-L405A, in addition to ABCG2-WT. The Automated Topology Builder and Repository (ATB)^34^ was used to develop united atom coordinates and GROMOS 54a7 compatable parameters for each drug at the predominant protonation state at physiological pH (7.0) (ATB repository molecule ID for erlotinib: 763850; Hoechst 33342: 29940; tariquidar: 1324). As previous structural studies suggest that ABCG2 operates via a peristalsis mechanism and does not form an occluded conformation that contains a stable, well-defined substrate binding space^7^, a single drug molecule of either erlotinib, Hoechst 33342 or tariquidar was docked into the TM pore of each ABCG2 model using Autodock Vina. After assessment of the docked poses against others with a score within 5 kJ/mol, the most energetically favorable scoring docked pose was used as the starting conformation for MDS^35^. Simulations were performed on the WT and each mutant ABCG2, in the presence and absence of docked substrate, giving 16 different systems in total. Each system was embedded in a 20% cholesterol and 80% POPC (1-palmitoyl-2-oleoyl-sn-glycero-3-phosphatidylcholine) bilayer and solvated using the simple point charge (SPC) explicit water model^36^, and 0.15 M of Na^+^ and Cl^−^ ions. Each system was energy minimized using a steepest descent algorithm and subsequently equilibrated with decreasing restraints on the protein backbone in six sequential 1 ns simulations (1000 kJ mol^–1^ nm^–2^, 500 kJ mol^–1^ nm^–2^, 100 kJ mol^–1^ nm^–2^, 50 kJ mol^–1^ nm^–2^, 10 kJ mol^–1^ nm^–2^, and 0 kJ mol^–1^ nm^–2^) using a 2 fs timestep. The coordinates from the final frame of each equilibrated system were used as the starting configuration for 300 ns production simulations, using an NPT thermodynamic ensemble. MDS use classical mechanics to calculate the time-evolution of the system to a local minimum-energy conformation. The position and orientation of all molecules in the simulation, including the docked compounds, changes throughout the simulation due to their thermal motion. To increase the statistical sampling of the ABCG2/substrate conformation space and ensure convergence of the docked compounds, non-biased production replicate (*n* = 3) simulations of each ABCG2 system were performed and a new random starting velocity was assigned at the start of each replicate simulation. In all simulations, the temperature was maintained at 300 K using the Bussi-Donadio-Parrinello velocity rescale thermostat and a coupling constant of 0.1 ps. Pressure was maintained at 1 bar using semi-isotropic pressure coupling using the Berendsen barostat with a 0.5 ps coupling constant and an isothermal compressibility of 4.5 × 10^–5^ bar^−1^. The length of covalent bonds was constrained using the LINCS algorithm^37^ implemented in GROMACS 2019.4. Electrostatic interactions were calculated using particle mesh Ewald and Van der Waals interactions were calculated with a cut-off of 1.2 nm.

### Analysis of simulation trajectories

Analysis of each system was performed on the *n* = 3 replicate 300 ns production simulations, to give a combined simulation time of 900 ns. Frames collected at 0.1 ns intervals. Data is reported as the mean ± standard deviation for the *n* = 3 production simulations, each lasting 300 ns. Simulations were analysed using the GROMACS 2019.4 toolkit, Visual Molecular Dynamics software (VMD) version 1.9.4^38^, and APBSmem (version 2.1.0)^39^ in conjunction with Chimera v1.17.1^40^. The APBS electrostatic potential maps were generated using the published settings outlined for the Step6p ABC trasporter^41^, with a 129 Å^3^ grid.

### Quantum mechanical (QM) calculations

All QM calculations of Tariquidar (taken from PDB ID 7NEQ) were carried out using Gaussian 16.3^42^. The geometry of tariquidar reported in PDB ID 7NEQ was optimized in the gas phase, at the M06-2X/6-31 + G(p) level of theory, followed by frequency calculations at the same level to confirm the nature of the calculated stationary points. An alternative geometry was optimized using the ATB (outlined above) followed by a single point energy calculation at M06-2X/6-31 + G(p) level of theory. All calculations are reported as gas phase energies (E) at the M06-2X/6-31 + G(p) level of theory.

### Data analysis and curve fitting

All experimental data analysis and curve fitting were carried out using GraphPad Prism (version 9.0.2). Melting temperature (T_m_) curve or dose-response curve (half maximal effective concentration (EC_50_) and half maximal inhibitory concentration (IC_50_)) were fit with the nonlinear regression with three parameters [1] or four parameters (variable slope) [2] and are shown in each figure according to the best-fit model as determined by *r*^*2*^ value.

1 $${Y}={{{{\rm{Bottom}}}}}+({{{{\rm{Top}}}}}-{{{{\rm{Bottom}}}}})/(1+{10}^{\wedge }(({{{{{\rm{LogEC}}}}}}_{50}-{X})))$$

2 $${Y}={{{{\rm{Bottom}}}}}+({{{{\rm{Top}}}}}-{{{{\rm{Bottom}}}}})/(1+{10}^{\wedge }(({{{{{\rm{LogEC}}}}}}_{50}-{X})\ast {HillSlope}))$$

Top and bottom are plateaus in the units of the *Y* axis.

Apparent EC_50_ and IC_50_ values were calculated from dose-response data using nonlinear regression analysis. All experiments were independently repeated at least three times. All *P* values were calculated using two-tailed Student’s *t* test.

### Reporting summary

Further information on research design is available in the Nature Portfolio Reporting Summary linked to this article.

## Supplementary information

Supplementary Information

Description of Additional Supplementary Files

Supplementary Data 1

Reporting Summary

## Source data

Source Data

## Data Availability

Structural data generated during this study are available on request from the corresponding author. Simulation parameter files, and the initial and final coordinates of the simulations are available at https://github.com/OMaraLab/ABCG2_N436A.  Source data are provided with this paper.
