# Publisher Correction: The net electrostatic potential and hydration of ABCG2 affect substrate transport

**DOI:** 10.1038/s41467-024-53357-4

**Published:** 2024-10-17

**Authors:** Tomoka Gose, Heather M. Aitken, Yao Wang, John Lynch, Evadnie Rampersaud, Yu Fukuda, Medb Wills, Stefanie A. Baril, Robert C. Ford, Anang Shelat, Megan L. O’Mara, John D. Schuetz

**Affiliations:** 1 Department of Pharmacy and Pharmaceutical Sciences, St. Jude Children’s Research Hospital, 262 Danny Thomas Place, Memphis, TN 38105 USA; 2 Australian Institute of Bioengineering and Nanotechnology, The University of Queensland, Australia, Cnr College Rd & Cooper Rd, St Lucia, QLD 4072 Australia; 3 Center for Applied Bioinformatics, St Jude Children’s Research Hospital, 262 Danny Thomas Place, Memphis, TN 38105 USA; 4 School of Biological Sciences, The University of Manchester, Oxford Road, Manchester, M13 9PL UK; 5 Department of Chemical Biology and Therapeutics, St. Jude Children’s Research Hospital, 262 Danny Thomas Place, Memphis, TN 38105 USA

Correction to: *Nature Communications* 10.1038/s41467-023-40610-5, published online 18 August 2023

In this article the author name Megan L. O’Mara was incorrectly written as Megan L. O’ Mara. The original article has been corrected.

