# Supplementary material for: The net electrostatic potential and hydration of ABCG2 affect substrate transport
Source: Nat Commun. 2023 Aug 18;14:5035. doi: 10.1038/s41467-023-40610-5 (PMC10439158; doi:10.1038/s41467-023-40610-5)
Supplement: Supplementary file 1 — Supplementary Information [file 41467_2023_40610_MOESM1_ESM.pdf]

Supplementary Information

The net electrostatic potential and hydration of ABCG2 affect substrate transport

Tomoka Gose[1], Heather M. Aitken[2], Yao Wang[1], John Lynch[1], Evadnie Rampersaud[3], Yu Fukuda[1], Medb Wills[1], Stefanie A. Baril[1], Robert C. Ford[4], Anang Shelat[5], Megan L. O'Mara[2], and John D. Schuetz[1]*

[1]Department of Pharmacy and Pharmaceutical Sciences, St. Jude Children's Research Hospital, 262 Danny Thomas Place, Memphis, TN 38105, USA

[2]Australian Institute of Bioengineering and Nanotechnology, The University of Queensland, Australia, Cnr College Rd & Cooper Rd, St Lucia, QLD 4072, Australia

[3]Center for Applied Bioinformatics, St Jude Children's Research Hospital, 262 Danny Thomas Place, Memphis, TN 38105, USA

[4]School of Biological Sciences, The University of Manchester, Oxford Road, Manchester M13 9PL, UK.

[5]Department of Chemical Biology and Therapeutics, St. Jude Children's Research Hospital, 262 Danny Thomas Place, Memphis, TN 38105, USA

*Corresponding author: John D. Schuetz

E-mail address: john.schuetz@stjude.org

Supplementary Information:

Supplementary Figures 1-12

Supplementary Tables 1-2

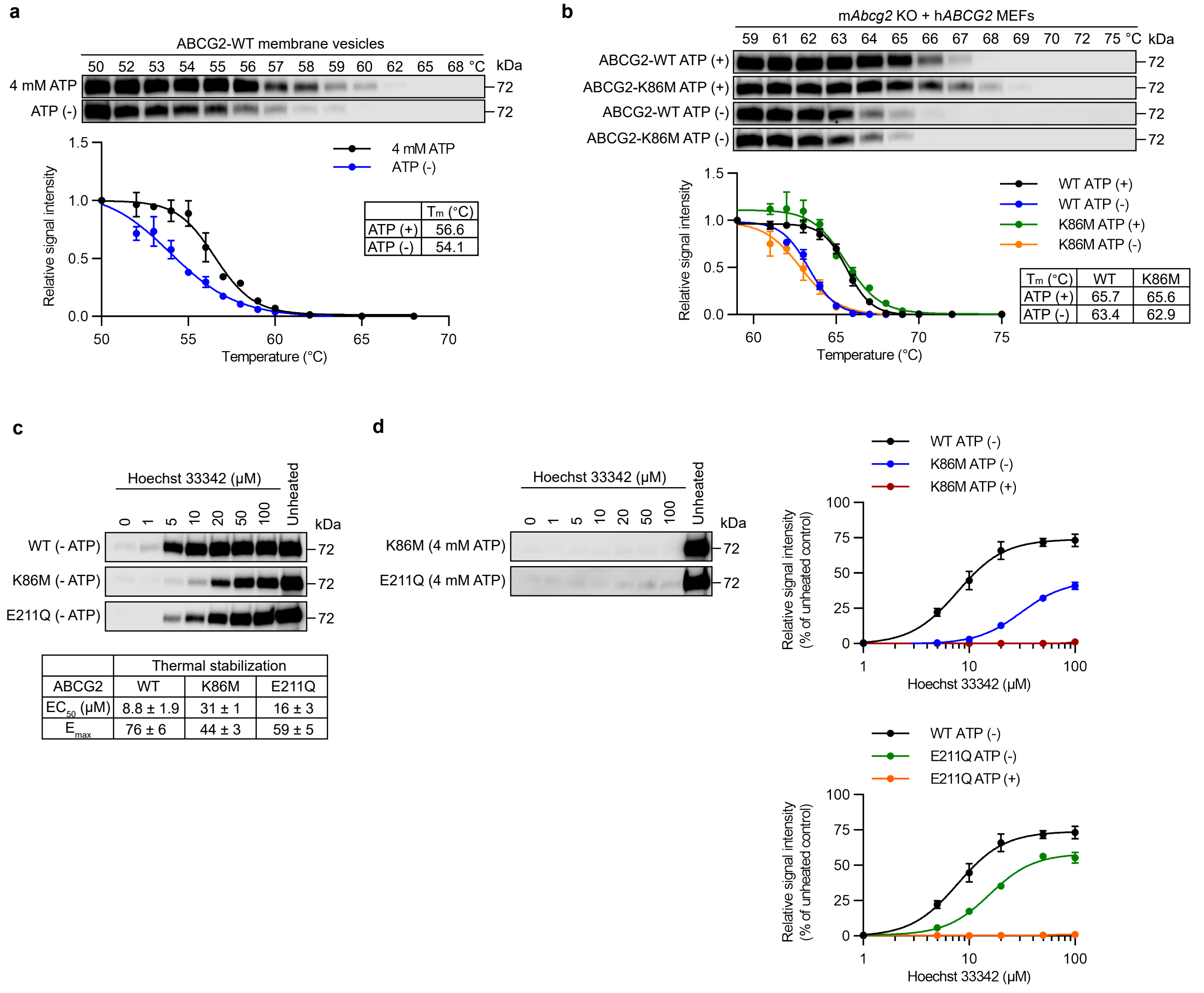

Supplementary Figure 1. Hoechst 33342 interaction with catalytically inactive ABCG2-K86M and ABCG2-E211Q mutants in the absence and presence of ATP.

a Melting temperature (T_m) curve of ABCG2-WT shifts upon ATP binding. The signal intensity was normalized to the intensity of the 50°C sample. Representative ABCG2 western blots are shown on the top side and densitometry quantification data from n = 3 independent experiments with SEM is shown on the bottom side. **b** For cellular depletion of ATP, *Abcg2*-KO MEFs expressing human ABCG2-WT or ABCG2-K86M were incubated in glucose-free D-MEM containing 50 mM 2-deoxy-d-glucose and 15 mM sodium azide for 30 min. The signal intensity was normalized to the intensity of the 59°C sample. Representative ABCG2 western blots are shown on the top side and densitometry quantification data from independent experiments (n = 7 (WT ATP (+)), n = 4 (K86M ATP (+)), or n = 3 (WT and K86M ATP (-))) with SEM is shown on the bottom side. **c** Hoechst 33342-induced thermal stabilization of catalytically inactive ABCG2-K86M and E211Q mutants in a concentration-dependent manner was observed in the absence of ATP. Representative ABCG2 western blots are shown on the top side and the EC_{50} and E_{max} values for Hoechst 33342 thermal stabilization in WT and these mutants are shown on the bottom side. **d** Hoechst 33342 was incapable of thermal stabilization of catalytically inactive ABCG2-K86M and E211Q mutants in the presence of 4 mM ATP. Representative ABCG2 western blots are shown on the left side and the densitometry quantification data (**c** and **d**) from independent experiments (n = 4 (WT) or n = 3 (K86M and E211Q)) with SEM are shown on the right side. Blot intensity is normalized to intensity from the unheated sample. Uncropped blots are provided as a Source data file.

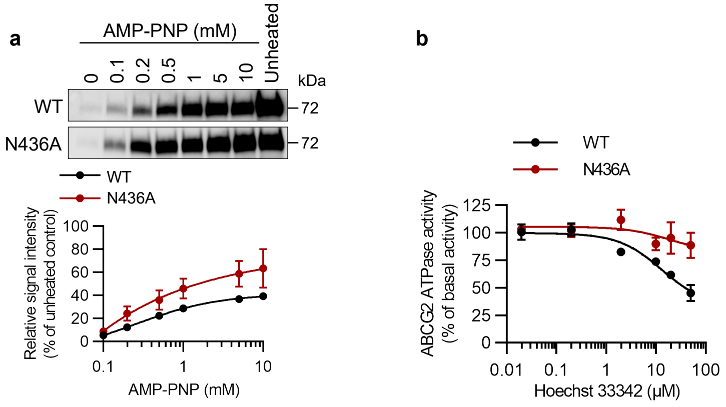

Supplementary Figure 2. N436A substitution affects nucleotide interaction and the inhibitory effect of Hoechst 33342 on the basal ABCG2-ATPase activity.

a Non-hydrolyzable ATP analog, AMP-PNP, stabilized both ABCG2-WT and ABCG2-N436A against thermal denaturation. Representative ABCG2 western blots are shown on the top side and densitometry quantification data from n = 3 independent experiments with SEM is shown on the bottom side. Uncropped blots are provided as a Source data file. **b** Effect of Hoechst 33342 on basal ABCG2 ATPase activity. Vanadate-sensitive ATPase activities of the plasma membranes from HEK293 cells expressing ABCG2-WT and ABCG2-N436A are quantified over a range of concentrations of Hoechst 33342. Data points represent the mean ± SEM from independent experiments (n = 4 (WT) or n = 6 (N436A)).

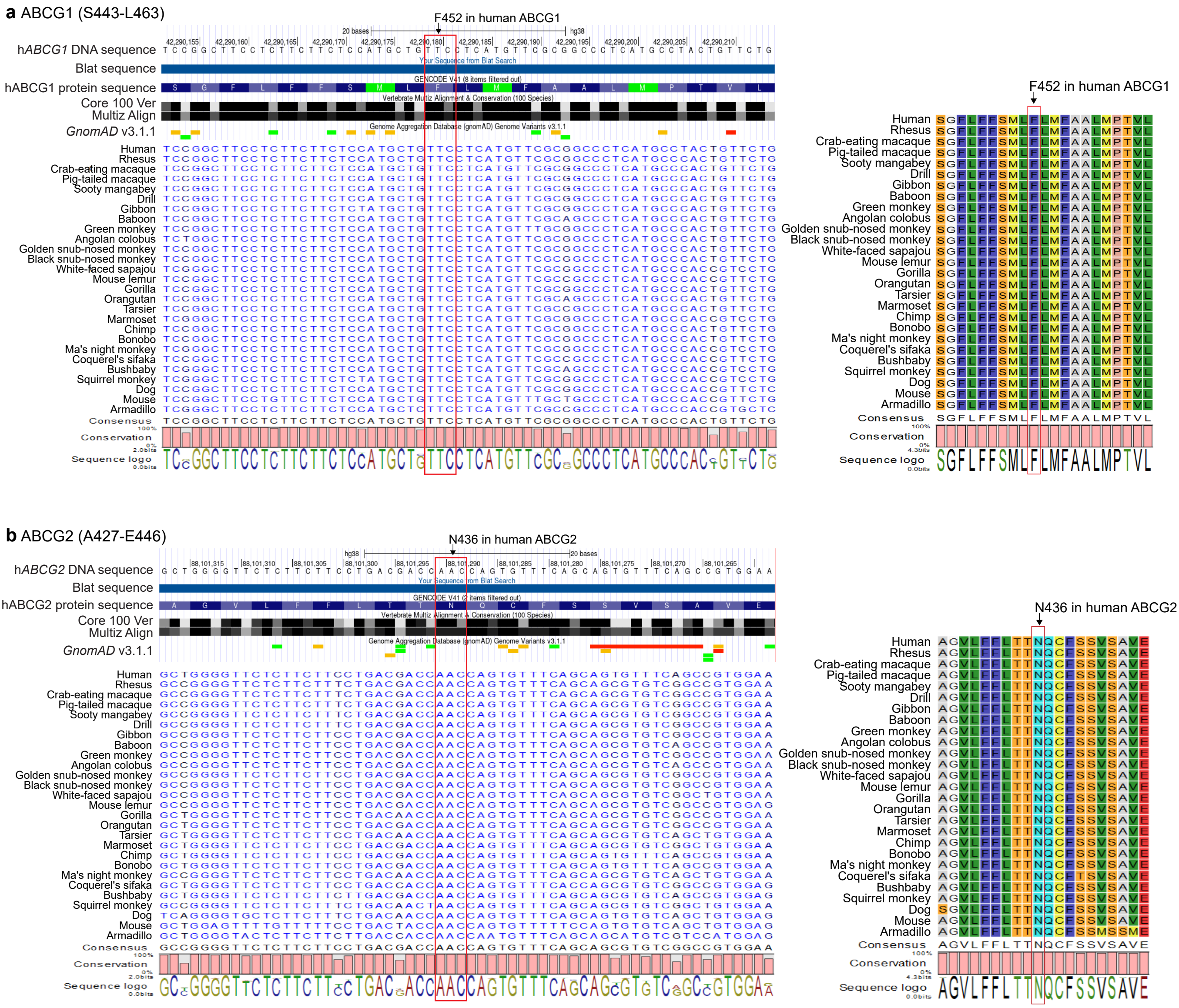

Supplementary Figure 3. Multiple sequence alignment of ABCG1 or ABCG2 across 27 mammals.
Multiple alignment of DNA (left) or protein (right) sequences across 27 mammals of the regions forming TMH2 in ABCG1 (**a**) or ABCG2 (**b**) and single nucleotide variants (SNVs) in the general population from Genome Aggregation Database (GnomAD v3.1.1) are shown. The position of N436 in human ABCG2 (corresponding to F452 in human ABCG1) is indicated by a red frame. The amino acid N436 of human ABCG2 protein is evolutionary conserved among 27 mammals.

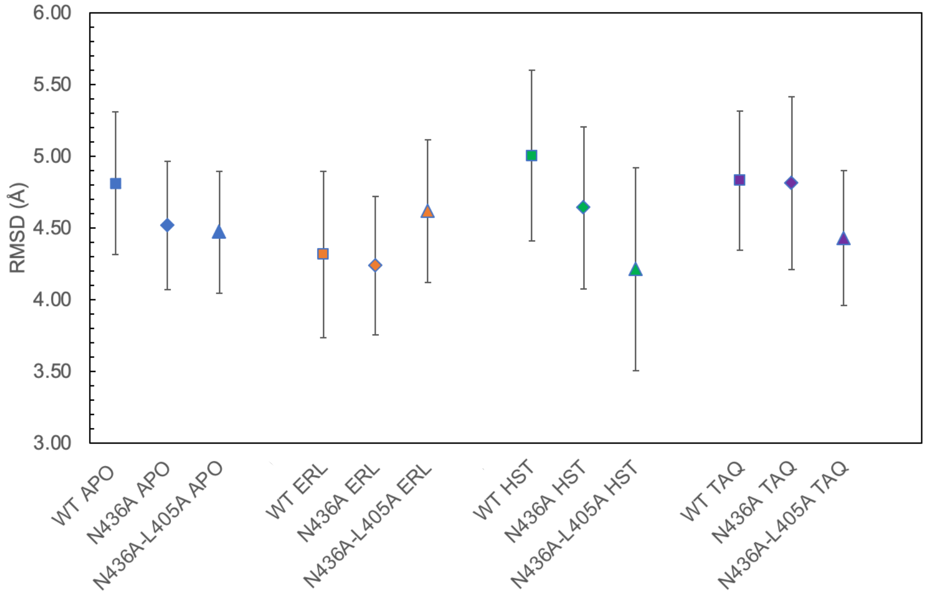

Supplementary Figure 4. Backbone RMSD of each ABCG2 conformation in the presence and absence of each compound.
Backbone RMSD of each ABCG2 conformation and compound combination reported in this study across combined 900ns of simulation for each system, which was obtained by concatenating the n = 3 replicate 300 ns simulations of each system. Data represents the mean ± S.D. ERL; erlotinib, HST; Hoechst 33342, TAQ; tariquidar. The time-dependent RMSD for each system is provided as a Source data file.

Residue	WT	N436A	N436A-L405A
GLN-393	65.3%	34.3%	42.0%
ALA-394	59.8%	83.3%	72.2%
GLN-398	47.9%	34.0%	35.5%
VAL-401	14.8%	1.7%	2.7%
THR-402	-	-	-
LEU-405	-	-	-
PHE-431	-	-	-
PHE-432	-	-	-
THR-435	-	-	-
ASN-436	-	-	-
PHE-439	69.5%	88.9%	**94.3%**
SER-440	35.6%	14.6%	22.2%
VAL-442	**94.7%**	88.6%	81.0%
SER-443	**95.5%**	72.9%	**94.0%**
GLU-446	**96.5%**	**93.6%**	**92.5%**
VAL-534	83.9%	83.5%	61.0%
SER-535	**97.2%**	**96.9%**	74.0%
THR-538	**99.5%**	**100.0%**	**96.0%**
LEU-539	**99.8%**	**99.3%**	85.5%
THR-542	**98.4%**	**99.6%**	**95.1%**
ILE-543	8.1%	8.6%	11.6%
VAL-546	1.2%	2.5%	4.2%

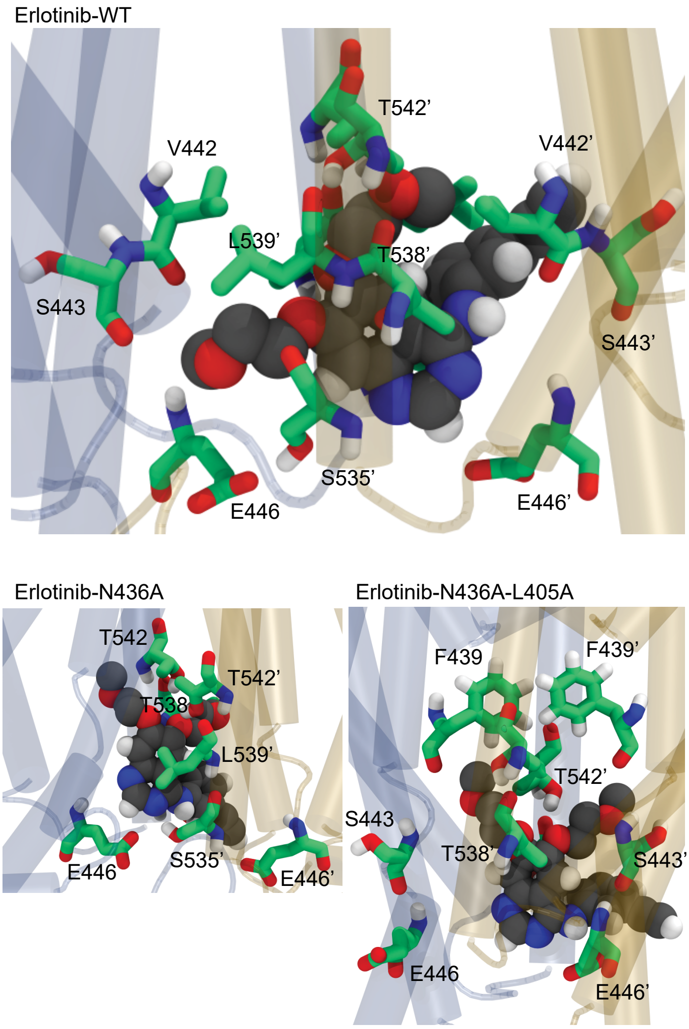

Supplementary Figure 5. Interactions between ABCG2 and erlotinib.
Left: Table of erlotinib-ABCG2 contact frequencies, measured as any atom of an amino acid that is located within 4 Å of any atom of erlotinib, across the n=3 replicate 300 ns simulations for each system. Right panels: MDS snapshots of the position of residues that are in contact with erlotinib (dark grey space fill) for 90% of the combined trajectories (green licorice) of ABCG3-WT (top), ABCG2-N436A (bottom left), ABCG2-N436A-L405A (bottom right). Unless otherwise indicated, all images are taken from the predominant conformation of the protein determined by cluster analysis of the combined trajectories, for each of ABCG2-WT, ABCG2-N436A, ABCG2-N436A-L405A.

Residue	WT	N436A	N436A-L405A
GLN-393	**96.9%**	48.4%	83.0%
ALA-394	21.5%	47.8%	8.8%
GLN-398	**94.1%**	54.7%	60.2%
VAL-401	82.6%	74.4%	84.2%
THR-402	64.1%	37.0%	46.6%
LEU-405	50.5%	52.4%	27.6%
PHE-431	-	0.07%	-
PHE-432	-	17.1%	12.5%
THR-435	-	12.7%	0.1%
ASN-436	**91.8%**	58.5%	47.1%
PHE-439	86.9%	63.0%	71.9%
SER-440	78.9%	65.9%	72.0%
VAL-442	**97.3%**	**90.5%**	**92.7%**
SER-443	**95.2%**	77.2%	52.4%
GLU-446	**99.9%**	**91.9%**	**98.6%**
VAL-534	89.1%	63.2%	72.2%
SER-535	**94.3%**	78.2%	77.0%
THR-538	**100.0%**	**94.6%**	**99.9%**
LEU-539	**94.6%**	**95.8%**	**96.6%**
THR-542	66.3%	75.7%	**98.6%**
ILE-543	58.5%	61.7%	66.1%
VAL-546	16.1%	58.6%	69.2%

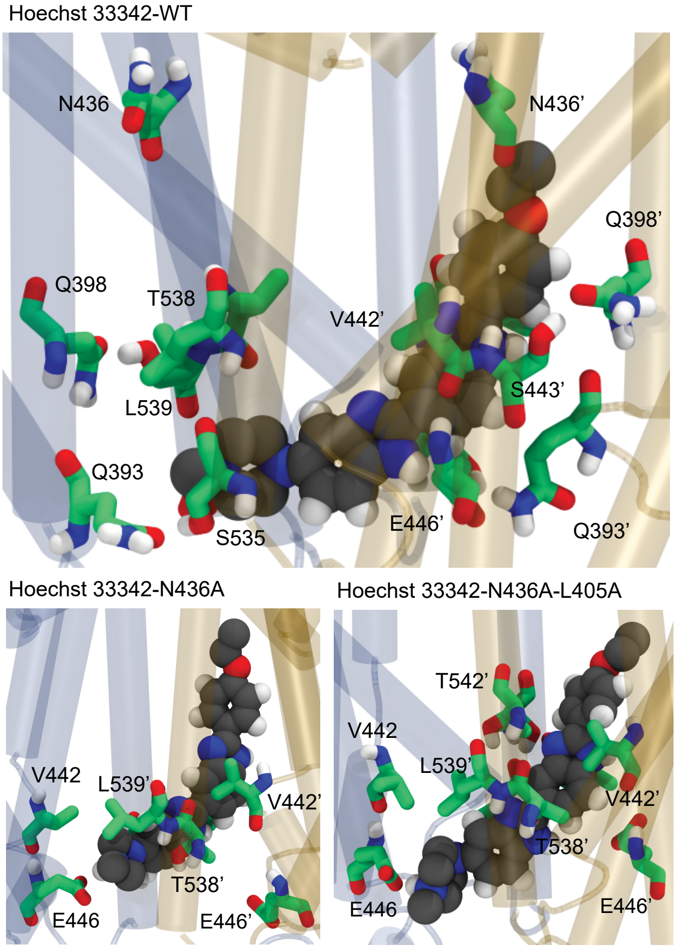

Supplementary Figure 6. Interactions between ABCG2 and Hoechst 33342.
Left: Table of Hoechst 33342-ABCG2 contact frequencies, measured as any atom of an amino acid that is located within 4 Å of any atom of Hoechst 33342, across the n=3 replicate 300 ns simulations for each system. Right: MDS snapshots of the position of residues that are in contact with Hoechst 33342 (dark grey space fill) for 90% of the combined trajectories (green licorice). Images are taken from the predominant conformation of the protein determined by cluster analysis of the combined trajectories, for each of ABCG2-WT, ABCG2-N436A, ABCG2-N436A-L405A.

Residue	WT	N436A	N436A-L405A
GLN-393	17.8%	26.8%	20.7%
ALA-394	15.7%	10.6%	13.6%
GLN-398	39.2%	33.9%	11.1%
VAL-401	79.0%	**92.2%**	**97.4%**
THR-402	74.2%	89.5%	68.8%
LEU-405	**96.1%**	**96.6%**	82.2%
PHE-431	40.77%	4.42%	56.91%
PHE-432	**98.3%**	58.8%	79.5%
THR-435	**91.7%**	32.0%	63.6%
ASN-436	**98.6%**	**95.3%**	**97.2%**
PHE-439	**100.0%**	**99.4%**	**100.0%**
SER-440	80.9%	87.7%	**90.9%**
VAL-442	**98.8%**	**99.7%**	80.4%
SER-443	82.0%	85.0%	53.0%
GLU-446	53.4%	41.9%	25.7%
VAL-534	57.3%	39.9%	16.9%
SER-535	86.5%	81.1%	39.8%
THR-538	**100.0%**	**99.8%**	45.1%
LEU-539	**99.8%**	**99.4%**	85.1%
THR-542	**100.0%**	**100.0%**	**100.0%**
ILE-543	74.7%	74.6%	88.6%
VAL-546	**98.6%**	**97.7%**	**99.9%**

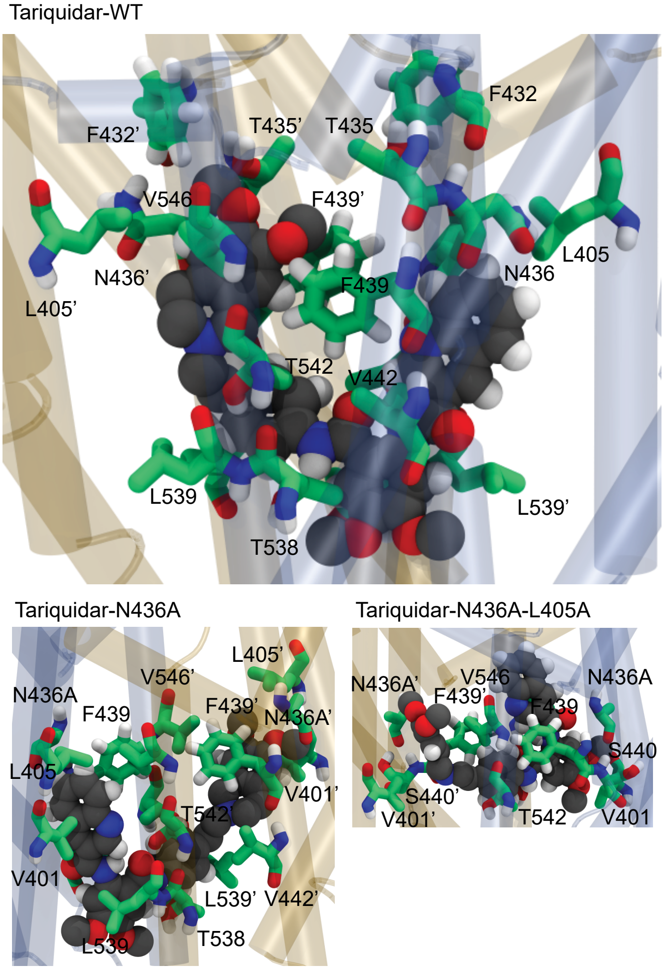

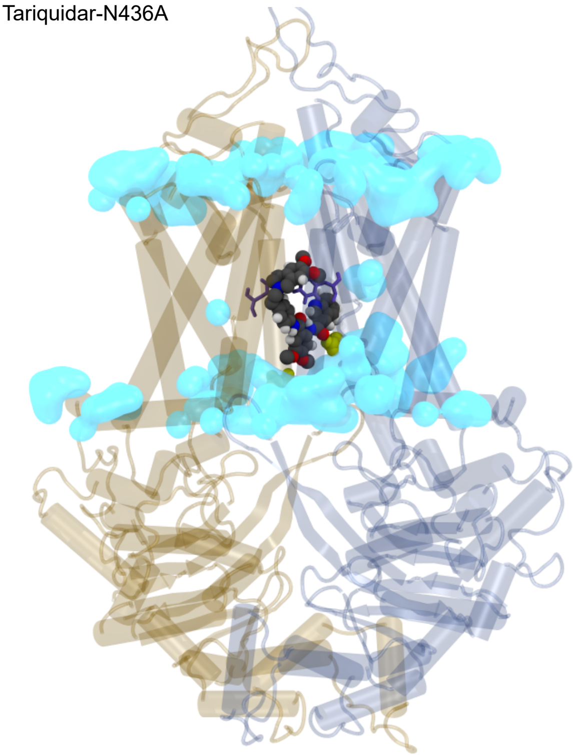

Supplementary Figure 7. Interactions between ABCG2 and tariquidar.
Top left: Table of tariquidar-ABCG2 contact frequencies, measured as any atom of an amino acid that is located within 4 Å of any atom of tariquidar, across the n=3 replicate 300 ns simulations for each system. Right: MDS snapshots of the position of residues that are in contact with tariquidar (dark grey space fill) for 90% of the combined trajectories (green licorice). Images are taken from the predominant conformation of the protein determined by cluster analysis of the combined trajectories, for each of ABCG2-WT, ABCG2-N436A, ABCG2-N436A-L405A. Bottom left: Transient snapshot of Tariquidar (dark grey space fill) bound structure in ABCG2-N436A. The location of water within 4 Å of the protein is shown in a cyan surface representation, and those waters within 4 Å of both the protein and tariquidar are shown in yellow-green space fill.

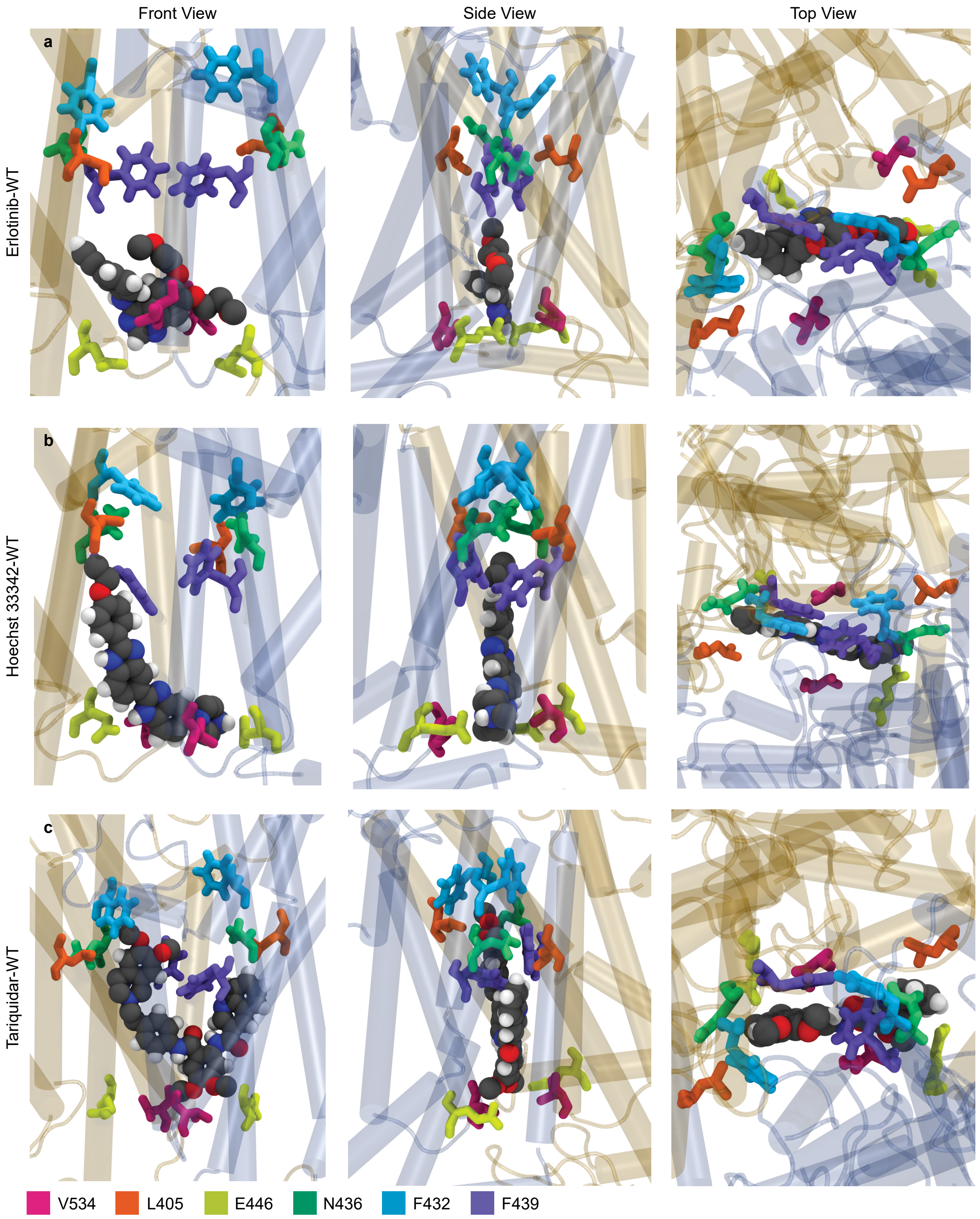

Supplementary Figure 8. ABCG2-WT MDS snapshots with ABCG2 ligands.
ABCG2-WT MDS snapshots of the position of the main residues discussed (N436 in green, L405 in orange, F439 in purple, F432 in blue, E446 in yellow, and V534 in magenta), from the front, side, and top view of the protein. Where necessary, the complex is rotated 180º to best show all relevant residues and the drug position (erlotinib (**a**), Hoechst 33342 (**b**), tariquidar (**c**); grey space fill). The N-terminal and C-terminal monomers are shown in blue and yellow respectively using a cartoon representation. Images are taken from the predominant conformation of the protein determined by cluster analysis.

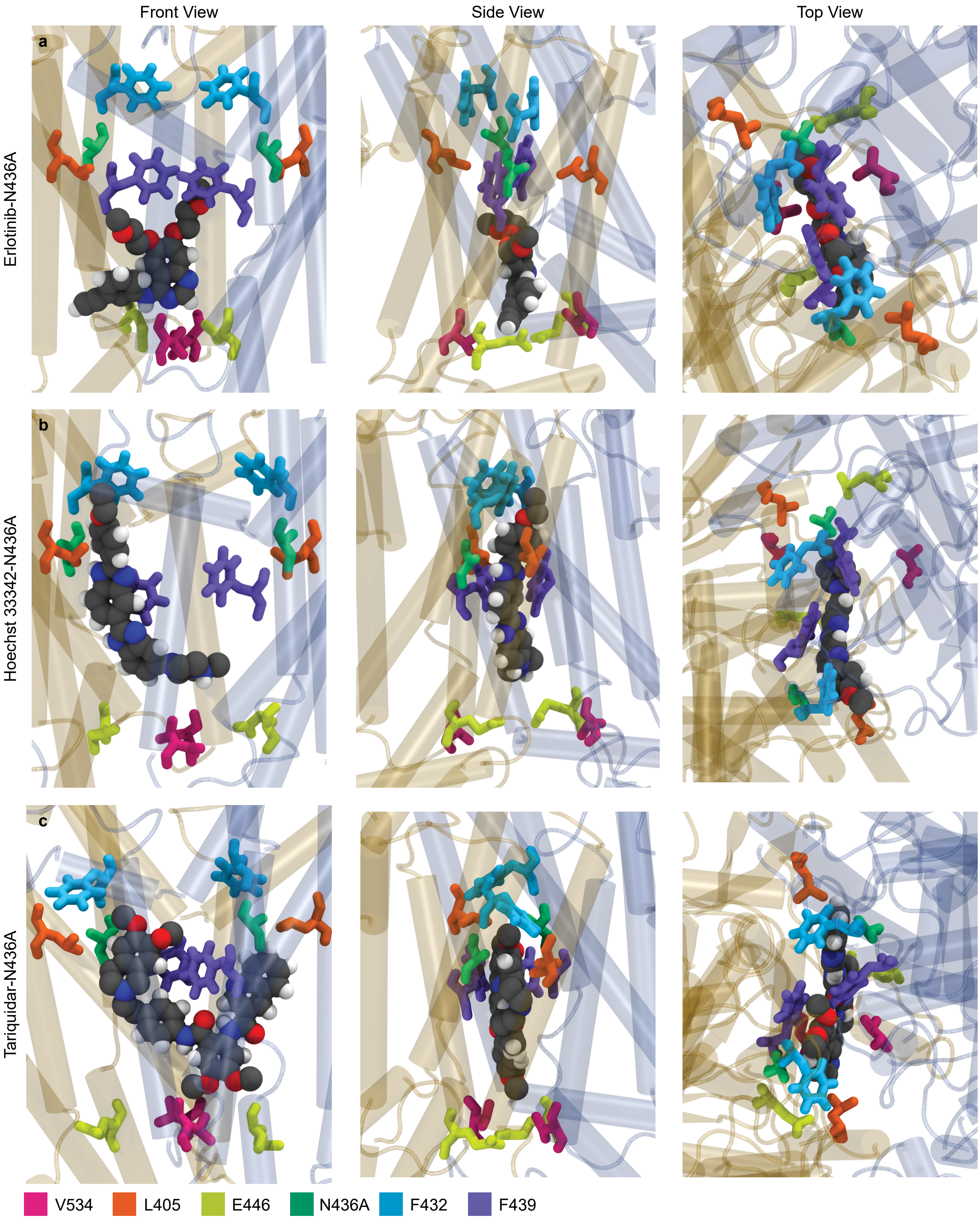

Supplementary Figure 9. ABCG2-N436A MDS snapshots with ABCG2 ligands.
ABCG2-N436A MDS snapshots of the position of the main residues discussed (N436A in green, L405 in orange, F439 in purple, F432 in blue, E446 in yellow, and V534 in magenta), taken from the front, side, and top view of the protein. Where necessary, the complex is rotated 180º to best show all relevant residues and the drug position (erlotinib (**a**), Hoechst 33342 (**b**), tariquidar (**c**); grey space fill). The N-terminal and C-terminal monomers are shown in blue and yellow respectively using a cartoon representation. Images are taken from the predominant conformation of the protein determined by cluster analysis.

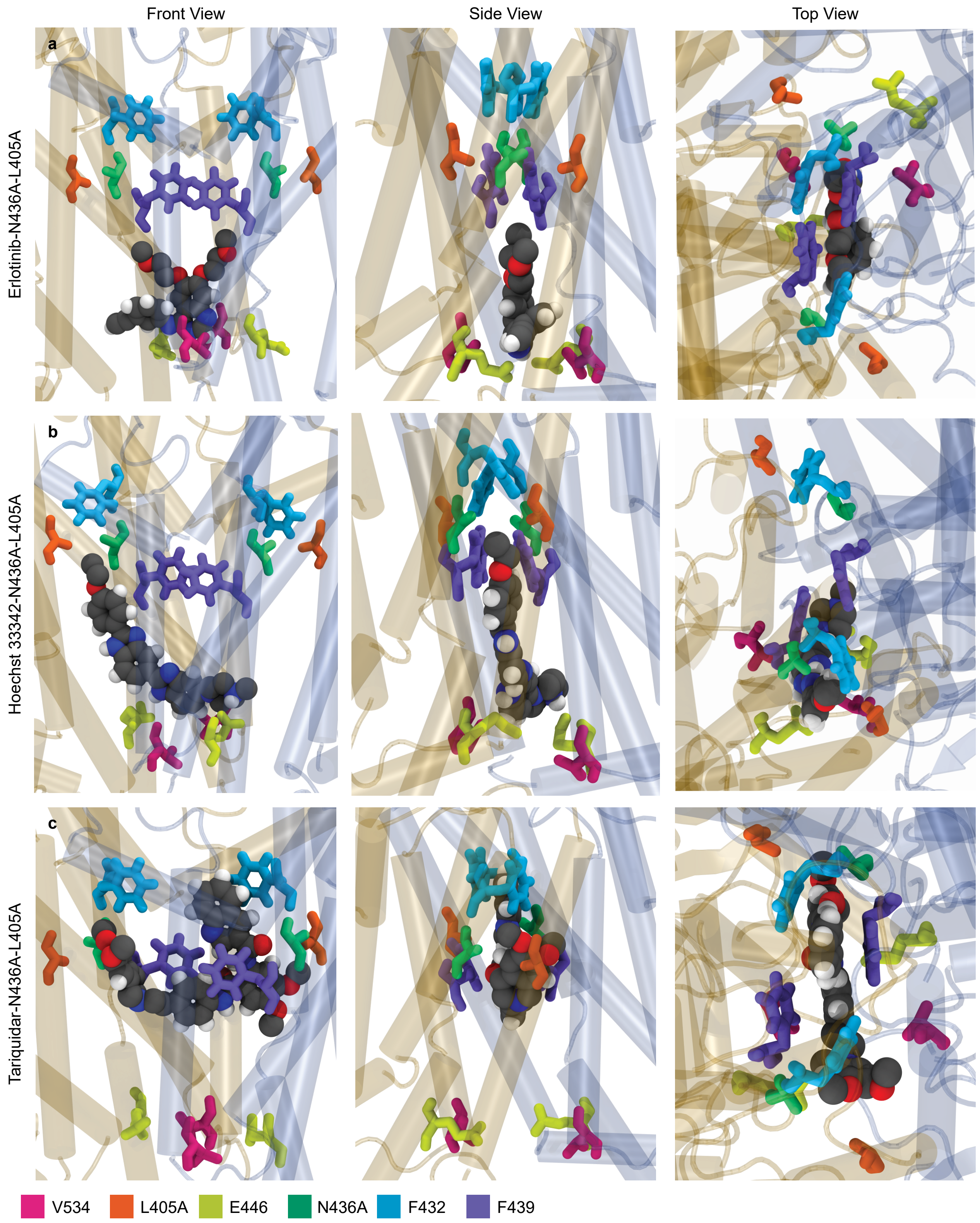

Supplementary Figure 10. ABCG2-N436A-L405A MDS snapshots with ABCG2 ligands.
ABCG2-N436A-L405A MDS snapshots of the position of the main residues discussed (N436A in green, L405A in orange, F439 in purple, F432 in blue, E446 in yellow, and V534 in magenta), taken from the front, side, and top view of the protein. Where necessary, the complex is rotated 180º to best show all relevant residues and the drug position (erlotinib (**a**), Hoechst 33342 (**b**), tariquidar (**c**); grey space fill). The N-terminal and C-terminal monomers are shown in blue and yellow respectively using a cartoon representation. Images are taken from the predominant conformation of the protein determined by cluster analysis.

a

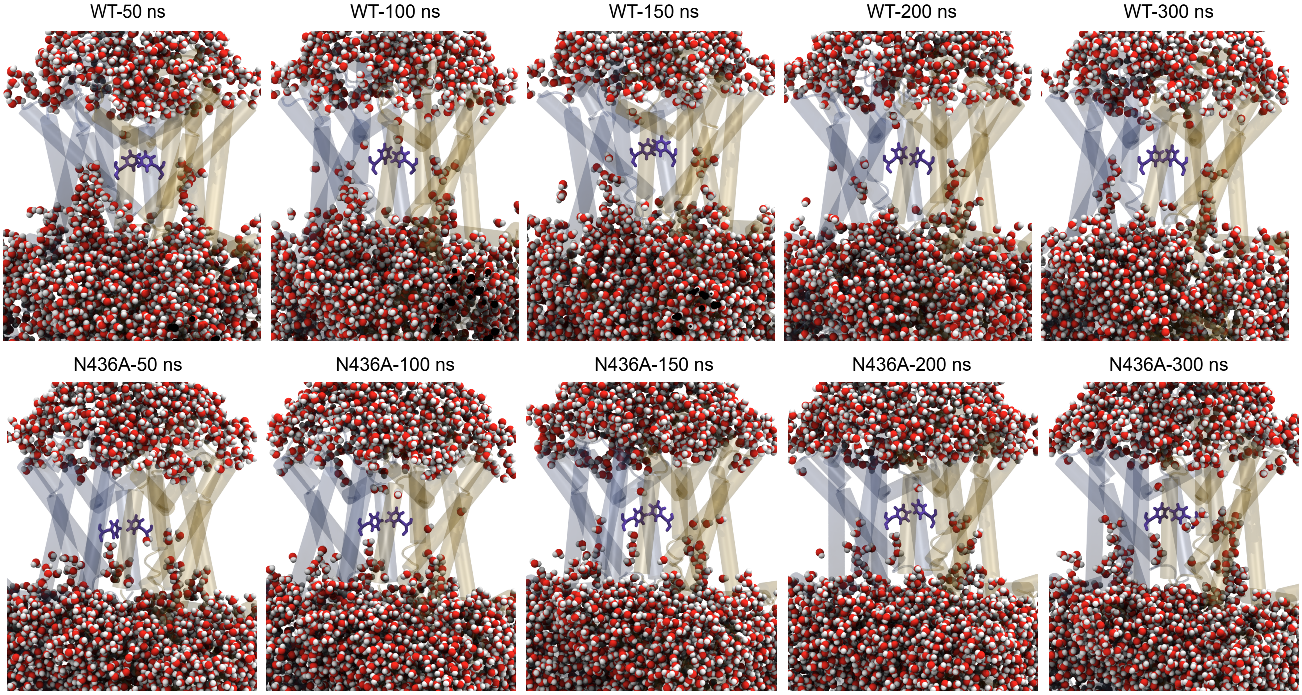

b

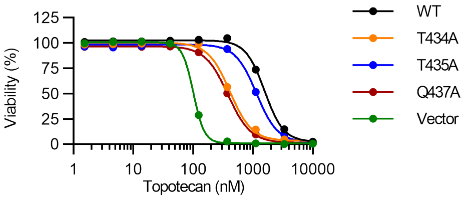

c

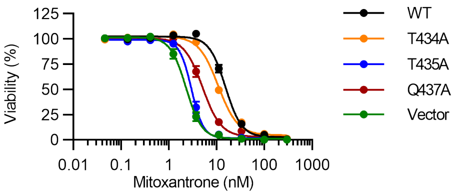

d

	Topotecan	Mitoxantrone
Volume	475	484
Van der waals surface area	60	104
LogP (o/w)	2.0	-0.5
H bond acceptor	5	6
H bond donor	2	6

Supplementary Figure 11. Alanine substitution of polar residues in the binding pocket of ABCG2 alters hydrophilic substrate transport.

a Water molecules within 4 Å of the protein are shown in red space fill after 50, 100, 150, 200, and 300 ns across a single replicate simulation for both ABCG2-WT and ABCG2-N436A. The cytotoxicity of topotecan (**b**) or mitoxantrone (**c**) was determined by CellTiter-Glo® assay using the *Abcg2*-KO MEFs expressing human ABCG2-WT (black), ABCG2-T434A (orange), ABCG2-T435A (blue), ABCG2-Q437A (red), or vector control (green). Data are presented as the mean ± SEM of 3 independent experiments conducted in duplicate. **d** The volume, Van der Waals surface area, logP, H-bond acceptor, and H-bond donor of topotecan and mitoxantrone were generated by molecular operating environment (MOE 2020.09). Topotecan and mitoxantrone were neutralized in MOE at pH 7.4 and 2D descriptors were computed from the resulting structures.

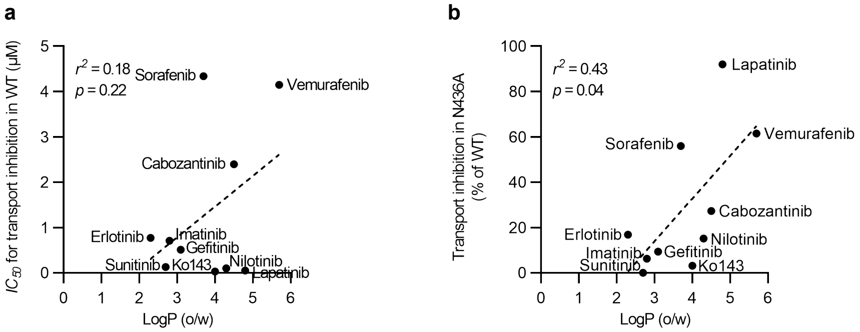

Supplementary Figure 12. Relationship between ABCG2 inhibitors' hydrophobicity (logP) and transport inhibition in ABCG2-WT or ABCG2-N436A.

a Relationship between logP values of ABCG2 inhibitors and the IC_{50} values for inhibition of Hoechst 33342 transport in ABCG2-WT. **b** Relationship between logP values of ABCG2 inhibitors and the effect of transport inhibition in ABCG2-N436A. R square and p value are shown.

	"Hydrophobic Gate"	
	F439-F439’	
	Mean (Å)	SD (Å)
**Apo-inward**		
WT	10.43	0.54
N436A	11.38	0.83
**Erlotinib**		
WT	12.43	1.07
N436A	11.62	0.64
**Hoechst 33342**		
WT	12.25	0.57
N436A	13.90	0.90
**Tariquidar**		
WT	12.91	0.50
N436A	13.51	0.61

Supplementary Table 1. Distance between residues F439-F439’ of opposite ABCG2 monomers.

Monomer-Monomer distance of key gating residue F439, measured as the distance between $C\alpha$ atoms of residue pairs across the combined 900 ns replicate simulations for each system.

	LogP (o/w)	Hoechst 33342 transport inhibition IC_{50} (μM)
Vemurafenib	5.7	4.15
Tariquidar	5.4	0.08
Lapatinib	4.8	0.05
Axitinib	4.8	6.04
Cabozantinib	4.5	2.40
Nilotinib	4.3	0.10
Ko143	4.0	0.03
Sorafenib	3.7	4.34
Gefitinib	3.1	0.51
Imatinib	2.8	0.71
Sunitinib	2.7	0.13
Erlotinib	2.3	0.77
Dasatinib	1.5	N/A

Supplementary Table 2. The logP values of ABCG2 inhibitors and the IC_{50} values for inhibition of Hoechst 33342 transport in ABCG2-WT.

The logP values were generated by molecular operating environment (MOE 2020.09). Compounds were neutralized in MOE at pH 7.4 and 2D descriptors were computed from the resulting structures.

N/A: not available.
