# Supplementary material for: The net electrostatic potential and hydration of ABCG2 affect substrate transport
Source: Nat Commun. 2023 Aug 18;14:5035. doi: 10.1038/s41467-023-40610-5 (PMC10439158; doi:10.1038/s41467-023-40610-5)
Supplement: Supplementary file 2 — Description of Additional Supplementary Files [file 41467_2023_40610_MOESM2_ESM.pdf]

Description of Additional Supplementary Files

File name: Supplementary Data 1

Description: Gaussian 16.3 output files for the gas-phase geometry optimization of tariquidar initiated from the conformation obtained from PDB ID 7NEQ, and from the docked conformation from MDS.
