# Supplementary material for: The net electrostatic potential and hydration of ABCG2 affect substrate transport
Source: Nat Commun. 2023 Aug 18;14:5035. doi: 10.1038/s41467-023-40610-5 (PMC10439158; doi:10.1038/s41467-023-40610-5)
Supplement: Supplementary file 3 — Supplementary Data 1 [file 41467_2023_40610_MOESM3_ESM.pdf]

Supplementary Data 1

Quantum mechanical (QM) calculations

Optimized Geometry – Tariquidar 7NEQ conformation

1\1\GINC-GADI-CPU-CLX-2441\FOpt\RM062X\6-31+G(d)\C38H36N4O6\HA0694\26-
Oct-2022\0\\#M062X/6-31+G* 6D INT(grid=ultrafine) IOP(2/17=4) Maxdisk=
200Gb OPT(maxstep=15)\\taq-7neq\\0,1\C,-4.9557824859,-1.2106909371,3.3
653912076\C,-1.9213224297,-0.8421849632,5.4298105169\C,0.0441889713,0.
6240464791,4.591023446\C,1.9160629413,-0.2109275207,3.2860448958\C,0.3
272109041,1.9021605685,4.1003889809\C,3.9829861622,2.3387355035,1.6161
55871\C,4.8598805772,2.212087576,0.4039388177\C,7.0961840388,2.2979616
455,-0.5224256482\C,5.2135638861,1.7057688279,-1.9464195654\C,-6.75782
4103,-1.4641058305,-1.463519381\C,-4.6698376352,-1.6631793537,-0.37357
10906\C,-3.26928097,-1.8270184438,-0.5043196126\C,-2.4783050673,-1.839
3233903,0.6373534725\C,-3.0468775355,-1.666330173,1.9032521724\C,-2.19
42555606,-1.7264844069,3.1472255452\C,-4.1068482485,-0.8969406038,4.36
48491753\C,-4.4212628108,-1.4882127876,2.0359049753\C,-5.2292905719,-1
.4986164922,0.8842055266\C,-1.0691867026,0.4198446119,5.5959216767\C,0
.8696011853,-0.4256861934,4.1752807548\C,2.1760229705,1.0728742902,2.7
980111386\C,1.3804364188,2.1411188988,3.2201200094\C,6.2284933663,2.45
89897849,0.5425643541\C,6.5834061673,1.8953875393,-1.7746887965\C,4.34
95230585,1.8684131084,-0.857268145\C,2.2212527563,1.1523286572,-1.9884
42246\C,0.7423605303,1.1856145491,-1.7458599469\C,0.1698677693,1.26323
62736,-0.4969587182\C,-1.2382438151,1.327104098,-0.3771081523\C,-1.895
5483163,1.4301161909,0.8772245996\C,-3.2634845152,1.5173584197,0.92915
46578\C,-4.0249176912,1.4919423697,-0.2675674409\C,-3.4161362951,1.375
3929985,-1.4934972314\C,-2.0035933836,1.2941116476,-1.5776319865\C,-0.
1197195615,1.1294927495,-2.8792752452\C,7.0285749171,1.2800329901,-4.0
212448278\C,8.9804888605,3.6263679707,-0.9576133795\C,-1.4201274647,-2
.1137196733,-1.9485143036\N,-2.7272748763,-0.8781860448,4.2151312676\N
,3.1902086424,1.2288006847,1.8292331973\N,2.950948951,1.7659509035,-1.
0027380956\N,-1.4256901811,1.1849293758,-2.8115276278\O,-5.3619929195,
-1.6436691526,-1.5408844493\O,3.9688574359,3.3193163416,2.3365853051\O
,2.7070883845,0.6352740242,-2.981996615\O,7.4939906137,1.7272702451,-2
.7597491571\O,8.4389360752,2.4643347687,-0.3362833153\O,-2.8090863113,
-1.9360937832,-1.7743025003\H,-6.0267906635,-1.1628286785,3.5284832128
\H,-1.292067221,-1.7444268602,5.4680563215\H,-2.5895945399,-0.90633206
02,6.2956346372\H,2.5447690355,-1.041775286,2.9743133579\H,-0.29406194
1,2.7390952626,4.4112526843\H,4.7945045732,1.4344011839,-2.904751773\H
,-7.1208585357,-1.486221244,-2.4909742657\H,-7.0098002161,-0.497865115
7,-1.0069730278\H,-7.2314804644,-2.2702229834,-0.8894762434\H,-1.40161
72841,-1.9550555563,0.5521863188\H,-1.177335211,-1.396696592,2.9155171
341\H,-2.1291391423,-2.7729412848,3.5137170847\H,-4.470512661,-0.58412
21774,5.3402136098\H,-6.2981458782,-1.3459207813,0.9952460227\H,-1.736
4181653,1.2890024414,5.57060429\H,-0.6318723607,0.3901090414,6.6044561
349\H,0.7028750237,-1.4333384698,4.5484915371\H,1.5855233059,3.1442601
676,2.8636680365\H,6.6300291787,2.7574555414,1.5066287091\H,0.76285785
13,1.2617274967,0.4189300798\H,-1.2994547101,1.4232574522,1.7891661431
\H,-3.769450397,1.5731679704,1.889107608\H,-5.1083923323,1.5453214234,
-0.2054167705\H,-3.9834650072,1.3238723963,-2.4173646794\H,0.329117588
1,1.0414025298,-3.8664863107\H,7.9169663216,1.1691208838,-4.6418789279
\H,6.3513398816,2.0135487156,-4.4730283337\H,6.5170027114,0.315658867,
-3.9299709852\H,10.0434399997,3.6337881535,-0.7140298556\H,8.503410558
2,4.5275130165,-0.5543033618\H,8.8496095238,3.5865899468,-2.0429074205
\H,-1.2529749392,-2.1360493981,-3.0250554403\H,-1.0865226471,-3.057817
49,-1.4983840774\H,-0.8518294923,-1.2819998276,-1.512360116\H,3.423125
9611,0.4063915018,1.2849806735\H,2.4098161636,2.2225854709,-0.28169609

29\\Version=ES64L-G16RevC.01\State=1-A\HF=-2139.2536416\RMSD=2.772e-09
\RMSF=5.137e-06\Dipole=-0.4728752,-0.3698781,0.9989619\Quadrupole=8.05
64366,-10.5795084,2.5230719,-3.2995005,-20.3213056,-8.3220674\PG=C01 [
X(C38H36N4O6)]\\@

Optimized Geometry – Tariquidar docked conformation

1\1\GINC-GADI-CPU-CLX-0068\SP\RM062X\6-31+G(d)\C38H38N4O6\HA0694\18-Oc
t-2022\0\\#M062X/6-31+G* 6D INT(grid=ultrafine) IOP(2/17=4) Maxdisk=20
0Gb\\taq-md\\0,1\H,0,7.187,-4.068,-4.186\C,0,6.907,-3.898,-3.136\C,0,7
.927,-4.088,-2.166\H,0,8.927,-4.508,-2.316\C,0,7.537,-3.878,-0.856\H,0
,8.167,-4.318,-0.066\C,0,6.287,-3.358,-0.426\C,0,5.987,-3.048,0.924\H,
0,6.847,-2.888,1.594\C,0,4.727,-2.538,1.184\C,0,3.727,-2.418,0.164\H,0
,2.697,-2.138,0.484\N,0,4.027,-2.738,-1.076\C,0,5.287,-3.178,-1.436\C,
0,5.647,-3.458,-2.796\H,0,5.007,-3.088,-3.616\C,0,4.497,-1.988,2.554\O
,0,5.457,-1.638,3.284\N,0,3.207,-1.598,2.884\H,0,2.607,-1.458,2.094\C,
0,2.537,-1.268,4.064\C,0,1.257,-0.658,4.094\C,0,0.747,-0.318,5.354\H,0
,-0.353,-0.318,5.364\C,0,1.307,-0.468,6.624\O,0,0.787,-0.228,7.874\C,0
,-0.583,0.132,8.104\C,0,2.567,-1.128,6.534\C,0,3.137,-1.518,5.334\H,0,
4.207,-1.768,5.354\O,0,3.367,-1.028,7.654\C,0,4.727,-1.418,7.874\C,0,0
.457,-0.228,2.914\O,0,0.987,-0.148,1.784\N,0,-0.763,0.402,3.174\H,0,-0
.883,0.642,4.134\C,0,-1.793,0.822,2.334\C,0,-1.903,0.622,0.944\H,0,-1.
113,0.082,0.394\C,0,-2.993,1.182,0.284\H,0,-3.023,1.032,-0.806\C,0,-4.
023,1.882,0.904\C,0,-3.873,2.122,2.264\H,0,-4.753,2.532,2.784\C,0,-2.8
13,1.562,2.964\H,0,-2.673,1.652,4.054\C,0,-5.193,2.262,0.064\C,0,-4.77
3,3.252,-1.026\N,0,-5.563,3.212,-2.256\C,0,-4.923,2.582,-3.416\C,0,-5.
593,2.732,-4.746\C,0,-5.003,2.222,-5.896\H,0,-4.163,1.512,-5.936\C,0,-
5.513,2.532,-7.156\O,0,-4.943,1.922,-8.266\C,0,-3.663,2.302,-8.766\C,0
,-6.643,3.382,-7.286\O,0,-7.033,3.722,-8.566\C,0,-7.943,4.812,-8.766\C
,0,-7.313,3.762,-6.126\H,0,-8.253,4.302,-6.246\C,0,-6.793,3.422,-4.886
\C,0,-7.463,3.852,-3.626\C,0,-6.463,4.322,-2.556\H,0,-0.74538,0.26551,
9.15315\H,0,-1.22195,-0.6454,7.74028\H,0,-0.80316,1.04431,7.59007\H,0,
5.00832,-1.17977,8.87849\H,0,5.36315,-0.89399,7.19163\H,0,4.82586,-2.4
7168,7.71628\H,0,-5.59685,1.38379,-0.39486\H,0,-5.92953,2.72542,0.6866
3\H,0,-4.84645,4.23918,-0.61981\H,0,-3.77939,2.9722,-1.3077\H,0,-3.940
88,2.99743,-3.50409\H,0,-4.96184,1.53273,-3.21003\H,0,-3.42249,1.70518
,-9.6209\H,0,-2.92437,2.15137,-8.00663\H,0,-3.68154,3.33463,-9.0457\H,
0,-8.13201,4.93142,-9.81238\H,0,-7.5136,5.71122,-8.37622\H,0,-8.86242,
4.60672,-8.25864\H,0,-8.02052,3.02656,-3.23521\H,0,-8.09811,4.68001,-3
.86252\H,0,-6.98911,4.61161,-1.67043\H,0,-5.90687,5.16348,-2.91312\\Ve
rsion=ES64L-G16RevC.01\State=1-A\HF=-2140.3119784\RMSD=2.663e-09\Dipol
e=-0.4199495,0.4287822,0.985165\Quadrupole=4.8717899,-7.7810586,2.9092
688,-30.8767634,-14.2084378,-11.3084967\PG=C01 [X(C38H38N4O6)]\\@
