# Supplementary material for: The net electrostatic potential and hydration of ABCG2 affect substrate transport
Source: Nat Commun. 2023 Aug 18;14:5035. doi: 10.1038/s41467-023-40610-5 (PMC10439158; doi:10.1038/s41467-023-40610-5)
Supplement: Supplementary file 4 — Reporting Summary [file 41467_2023_40610_MOESM4_ESM.pdf]

Corresponding author(s): John D. Schuetz
Last updated by author(s): Jun 30, 2023

Reporting Summary

Nature Portfolio wishes to improve the reproducibility of the work that we publish. This form provides structure for consistency and transparency in reporting. For further information on Nature Portfolio policies, see our Editorial Policies and the Editorial Policy Checklist.

Statistics

For all statistical analyses, confirm that the following items are present in the figure legend, table legend, main text, or Methods section.

n/a	Confirmed	
☐	☒	The exact sample size (*n*) for each experimental group/condition, given as a discrete number and unit of measurement
☐	☒	A statement on whether measurements were taken from distinct samples or whether the same sample was measured repeatedly
☐	☒	The statistical test(s) used AND whether they are one- or two-sided<br>*Only common tests should be described solely by name; describe more complex techniques in the Methods section.*
☒	☐	A description of all covariates tested
☒	☐	A description of any assumptions or corrections, such as tests of normality and adjustment for multiple comparisons
☐	☒	A full description of the statistical parameters including central tendency (e.g. means) or other basic estimates (e.g. regression coefficient) AND variation (e.g. standard deviation) or associated estimates of uncertainty (e.g. confidence intervals)
☐	☒	For null hypothesis testing, the test statistic (e.g. *F*, *t*, *r*) with confidence intervals, effect sizes, degrees of freedom and *P* value noted<br>*Give P values as exact values whenever suitable.*
☒	☐	For Bayesian analysis, information on the choice of priors and Markov chain Monte Carlo settings
☒	☐	For hierarchical and complex designs, identification of the appropriate level for tests and full reporting of outcomes
☒	☐	Estimates of effect sizes (e.g. Cohen's *d*, Pearson's *r*), indicating how they were calculated

Our web collection on statistics for biologists contains articles on many of the points above.

Software and code

Policy information about availability of computer code

Data collection	Western Blotting - Odyssey Fc Imaging System (LI-COR, Image Studio 5.2)<br>Measurement of luminescence (CellTiter-Glo), fluorescence, and absorbance - BioTek Synergy™ H4<br>Measurement of [14C]-labeled erlotinib radioactivity - Liquid scintillation counter<br>Flow cytometry - BD LSRFortessa flow cytometer (BD Biosciences)<br>Molecular Dynamics Simulations - Groningen Machine for Chemical Simulation engine, version 2019.4 (GROMACS 2019.4)
Data analysis	GraphPad Prism (version 9.0.2 for Windows), Image Studio Lite (LI-COR, version 5.2), Molecular Operating Environment (MOE 2020.09), Groningen Machine for Chemical Simulation engine, version 2019.4 (GROMACS 2019.4), Visual Molecular Dynamics software (VMD) version 1.9.4, APBSmem (version 2.1.0), Chimera v1.17.1, FlowJo v10

For manuscripts utilizing custom algorithms or software that are central to the research but not yet described in published literature, software must be made available to editors and reviewers. We strongly encourage code deposition in a community repository (e.g. GitHub). See the Nature Portfolio guidelines for submitting code & software for further information.

Data

Policy information about availability of data

All manuscripts must include a data availability statement. This statement should provide the following information, where applicable:

- Accession codes, unique identifiers, or web links for publicly available datasets
- A description of any restrictions on data availability
- For clinical datasets or third party data, please ensure that the statement adheres to our policy

Structural data referenced in this study are available in the Protein Data Bank with the accession numbers 6ETI and 7NEQ. Structural data generated during this study are available on reasonable request from the corresponding author. Simulation parameter files, and the initial and final coordinates of the simulations are available at https://github.com/OMaraLab/ABCG2_N436A. A reporting summary for this article is available as a supplementary information file. Source data underlying Figs. 1-5, Supplementary Figs. 1, 2, 4, 11, 12, and Supplementary Table 1 are provided as a Source data file.

Research involving human participants, their data, or biological material

Policy information about studies with human participants or human data. See also policy information about sex, gender (identity/presentation), and sexual orientation and race, ethnicity and racism.

Reporting on sex and gender	N/A
Reporting on race, ethnicity, or other socially relevant groupings	N/A
Population characteristics	N/A
Recruitment	N/A
Ethics oversight	N/A

Note that full information on the approval of the study protocol must also be provided in the manuscript.

Field-specific reporting

Please select the one below that is the best fit for your research. If you are not sure, read the appropriate sections before making your selection.

☒ Life sciences ☐ Behavioural & social sciences ☐ Ecological, evolutionary & environmental sciences

For a reference copy of the document with all sections, see nature.com/documents/nr-reporting-summary-flat.pdf

Life sciences study design

All studies must disclose on these points even when the disclosure is negative.

Sample size	No sample size calculation was performed. Sample size was chosen based on the nature of each experiment. At least three biologically independent replicates were performed in all experiments. Each single data point was represented as a grey dot in the graphs. Sample size for each experiment is indicated in all figure legends.
Data exclusions	No data were excluded from analyses.
Replication	All experiments were repeated at least three times independently. All attempts at replication were successful. The number of replicates is indicated in the figure legends.
Randomization	Randomization was not applicable. Experiments were not randomized but the data was validated by replication.
Blinding	It is not applicable to studies reported. Investigators were not blinded to the samples that were compared against a positive control. All data was validated by replication.

Reporting for specific materials, systems and methods

We require information from authors about some types of materials, experimental systems and methods used in many studies. Here, indicate whether each material, system or method listed is relevant to your study. If you are not sure if a list item applies to your research, read the appropriate section before selecting a response.

Materials & experimental systems

n/a	Involved in the study	
☐	☒	Antibodies
☐	☒	Eukaryotic cell lines
☒	☐	Palaeontology and archaeology
☒	☐	Animals and other organisms
☒	☐	Clinical data
☒	☐	Dual use research of concern
☒	☐	Plants

Methods

n/a	Involved in the study	
☒	☐	ChIP-seq
☐	☒	Flow cytometry
☒	☐	MRI-based neuroimaging

Antibodies

Antibodies used

ABCG2 antibody (rat monoclonal, BXP-53, Enzo Life Sciences, Cat# ALX-801-036-C100, 1:500 dilution), HRP-conjugated secondary antibody raised against rat IgG (polyclonal, Jackson ImmunoResearch Laboratories, Code 712-035-150, 1:10,000 dilution), ATP1A1 antibody (mouse monoclonal, Novus Biologicals, Cat# NB300-146SS, 1:5,000 dilution), HRP-conjugated secondary antibody raised against mouse IgG (polyclonal, Jackson ImmunoResearch Laboratories, Code 715-035-150, 1:10,000 dilution), ABCG2 antibody (mouse monoclonal, 5D3, Santa Cruz Biotechnology, Cat# sc-18841, final concentration 0.5 μg/mL), anti-mouse IgG-Alexa Fluor 647 (polyclonal, Thermo Fisher Scientific, Cat# A21235, 1:100 dilution)

Validation

All commercial antibodies were validated according to the companies.
ABCG2 antibody (rat monoclonal, BXP-53, Enzo Life Sciences) - WB validated for human and mouse. We have validated using positive and negative controls (hABCG2-overexpressing and mAbcg2-knockout cells).
https://www.enzolifesciences.com/ALX-801-036/breast-cancer-resistance-protein-monoclonal-antibody-bxp-53/
ATP1A1 antibody (mouse monoclonal, Novus Biologicals) - WB validated for Human, Mouse, Rat, Porcine, Bovine, Canine, Drosophilia, Guinea Pig, Primate, Rabbit, Sheep, Xenopus, and Yeast.
https://www.novusbio.com/products/sodium-potassium-atpase-alpha-1-antibody-4646_nb300-146
ABCG2 antibody (mouse monoclonal, 5D3, Santa Cruz Biotechnology) - Zhou, S., Schuetz, J. D., Bunting, K. D., Colapietro, A. M., Sampath, J., Morris, J. J., Lagutina, I., Grosveld, G. C., Osawa, M., Nakauchi, H., and Sorrentino, B. P. (2001) Nat. Med. 7, 1028–1034
https://www.scbt.com/p/abcg2-antibody-5d3
HRP-conjugated secondary antibodies raised against rat IgG or mouse IgG (Jackson ImmunoResearch Laboratories)
https://www.jacksonimmuno.com/catalog/products/712-035-150
https://www.jacksonimmuno.com/catalog/products/715-035-150
Anti-mouse IgG-Alexa Fluor 647 (polyclonal, Thermo Fisher Scientific)
https://www.thermofisher.com/antibody/product/Goat-anti-Mouse-IgG-H-L-Cross-Adsorbed-Secondary-Antibody-Polyclonal/A-21235

Eukaryotic cell lines

Policy information about cell lines and Sex and Gender in Research

Cell line source(s)

Mouse embryonic fibroblast (MEF) cell line was derived from Abcg2-KO mice that were obtained from the Sorrentino lab (S Zhou et al., PNAS. 2002 PMID: 12218177). HEK293 cells were received from collaborators 25 years ago.

Authentication

We have done authentication for MEF cells by STR analyses. HEK293 cells were not authenticated.

Mycoplasma contamination

All cell lines have been tested to be mycoplasma negative.

Commonly misidentified lines
(See ICLAC register)

No commonly misidentified lines were involved in this study.

Flow Cytometry

Plots

Confirm that:

☒ The axis labels state the marker and fluorochrome used (e.g. CD4-FITC).

☒ The axis scales are clearly visible. Include numbers along axes only for bottom left plot of group (a 'group' is an analysis of identical markers).

☒ All plots are contour plots with outliers or pseudocolor plots.

☒ A numerical value for number of cells or percentage (with statistics) is provided.

Methodology

Sample preparation

Cells were grown on plates, trypsinized, and then stained for flow-cytometry.

Instrument	BD LSRFortessa flow cytometer (BD Biosciences)
Software	FlowJo v10
Cell population abundance	N/A
Gating strategy	Cells were gated first on FSC-A/SSC-A (cell size), second on FSC-A/PI (live /dead cell exclusion), and third on FSC-A/FSC-H (singlets). Gating strategy is provided as a Source Data file.

☒ Tick this box to confirm that a figure exemplifying the gating strategy is provided in the Supplementary Information.
